# Evaluation of mosquito electrocuting traps as a safe alternative to the human landing catch for measuring human exposure to malaria vectors in Burkina Faso

**DOI:** 10.1186/s12936-019-3030-5

**Published:** 2019-12-02

**Authors:** Antoine Sanou, W. Moussa Guelbéogo, Luca Nelli, K. Hyacinth Toé, Soumanaba Zongo, Pierre Ouédraogo, Fatoumata Cissé, Nosrat Mirzai, Jason Matthiopoulos, N’falé Sagnon, Heather M. Ferguson

**Affiliations:** 1 Institute of Biodiversity, Animal Health and Comparative Medicine, University of Glasgow, Graham Kerr Building, Glasgow, G12 8QQ UK; 2 Centre National de Recherche et de Formation sur le Paludisme, Av. Kunda nyooré, PO Box 2208, Ouagadougou, Burkina Faso; 3 Bioelectronics Units, University of Glasgow, Graham Kerr Building, Glasgow, G12 8QQ UK

**Keywords:** Mosquito electrocuting trap, Human landing trap, Malaria, *An. gambiae*, Host-seeking behaviour, Outdoor biting

## Abstract

**Background:**

Measuring human exposure to mosquito bites is a crucial component of vector-borne disease surveillance. For malaria vectors, the human landing catch (HLC) remains the gold standard for direct estimation of exposure. This method, however, is controversial since participants risk exposure to potentially infected mosquito bites. Recently an exposure-free mosquito electrocuting trap (MET) was developed to provide a safer alternative to the HLC. Early prototypes of the MET performed well in Tanzania but have yet to be tested in West Africa, where malaria vector species composition, ecology and behaviour are different. The performance of the MET relative to HLC for characterizing mosquito vector population dynamics and biting behaviour in Burkina Faso was evaluated.

**Methods:**

A longitudinal study was initiated within 12 villages in Burkina Faso in October 2016. Host-seeking mosquitoes were sampled monthly using HLC and MET collections over 14 months. Collections were made at 4 households on each night, with METs deployed inside and outside at 2 houses, and HLC inside and outside at another two. Malaria vector abundance, species composition, sporozoite rate and location of biting (indoor *versus* outdoor) were recorded.

**Results:**

In total, 41,800 mosquitoes were collected over 324 sampling nights, with the major malaria vector being *Anopheles gambiae* sensu lato (s.l.) complex. Overall the MET caught fewer *An. gambiae* s.l. than the HLC (mean predicted number of 0.78 *versus* 1.82 indoors, and 1.05 *versus* 2.04 outdoors). However, MET collections gave a consistent representation of seasonal dynamics in vector populations, species composition, biting behaviour (location and time) and malaria infection rates relative to HLC. As the relative performance of the MET was somewhat higher in outdoor *versus* indoor settings, this trapping method slightly underestimated the proportion of bites preventable by LLINs compared to the HLC (MET = 82.08%; HLC = 87.19%).

**Conclusions:**

The MET collected proportionately fewer mosquitoes than the HLC. However, estimates of *An. gambiae* s.l. density in METs were highly correlated with HLC. Thus, although less sensitive, the MET is a safer alternative than the HLC. Its use is recommended particularly for sampling vectors in outdoor environments where it is most sensitive.

## Background

Measurement of malaria transmission and evaluation of vector control requires estimation of human exposure to malaria-infected mosquitoes [1]. This exposure is often estimated in terms of the Entomological Inoculation Rate (EIR [2]) defined as the mean number of malaria-infected mosquito bites a person would be expected to receive in a given setting [1, 3]. Accurate estimation of exposure to mosquito bites is crucial for evaluating interventions, thus there is an urgent need for reliable and robust methods to give unbiased estimates of exposure in a range of settings [3]. Several methods have been used to measure mosquito host-seeking behaviour and human exposure to mosquitoes. Historically, the human landing catch (HLC) has been the most commonly used method for African malaria vectors and is considered a gold standard approach for direct measurement of human-mosquito contact in both indoors and outdoors settings [4]. In this method, human volunteers expose part of their body, usually the lower legs, to lure host-seeking mosquitoes that are then collected upon landing [4].

Although HLC provides a direct measurement of human exposure to bites, its estimates can be biased due to variation in the skill of mosquito collectors and their attractiveness to mosquitoes [5–8]. HLC also raise ethical concerns as collectors are exposed to potentially infectious mosquito bites [9]. While this risk can be minimized by providing malaria prophylaxis to collectors, protection cannot be guaranteed in areas of drug resistance or where mosquitoes are carrying other pathogens, such as arboviruses [10, 11]. One African study indicated that HLC participants had no increased risk of malaria [12], but there remains a concerns about disease exposure in areas where other mosquito-borne pathogens are circulating.

Due to these limitations of the HLC, a range of alternative “exposure-free” methods have been developed. Most common is the CDC light trap [4, 13–15], a trap that can be placed next to a person sleeping under a bed-net and used to collect mosquitoes that would have otherwise have fed on them [14]. Although effective and easy to use in indoor environments [16], this method is harder to implement outdoors and may not accurately reflect human exposure in this setting [16–18]. Furthermore, CDC light catches can be affected by variation in the trap-light intensity [19, 20] and colour [16]. Other “exposure-free” methods include the human-baited double net trap (HDN) [18], Suna Trap [21], Host Decoy Trap (HDT; [22]), Ifakara tent trap design C (ITT-C) [23] and the Mbita trap [11]. Of these the last two have the same limitation as the CDC light trap of not being suitable or representative for measuring exposure in outdoor environments. For example, the tent trap only samples mosquitoes that are capable of entering a small enclosed structure, therefore, disproportionately catches indoor biting mosquito species [24]. The HDN was as efficient as the HLC in collecting outdoor anthropophilic mosquito. However, like the Tent Trap, it may also be selectively biased towards indoor biting mosquitoes, or sample vectors that enter the net to rest instead of biting [18, 25]. Similarly the Mbita trap had poor performance relative to the HLC in a setting where most vectors were exophilic and zoophilic [26]. Both the SUNA and Host Decoy Trap have shown promise for sampling outdoor biting malaria vectors [21, 22]; although may under [27] or overestimate [22] human exposure relative to the HLC. Given the growing recognition of outdoor biting as a major source of residual transmission in Africa [28–30] there is a clear need for improved methods that can reliably and safely measure exposure outside of homes.

The mosquito electrocuting trap (MET) has been developed as a representative and safer alternative method to the HLC for measuring human exposure to mosquito vectors both indoors and outdoors [17, 31, 32]. As previously described [31], the MET builds on previous work using electrified nets and grids to trap flies [33, 34] and mosquitoes [35–39] attracted to hosts or their odours. This trap consists of four panels that can be assembled into a box around the lower legs of seated human [17, 31] (Additional file 1: Figure S1), or an entire host (human or cow) [32]. Each panel consists of an electrified surface that allows free air movement and is safe to use in close proximity to a human volunteer, and intercepts and kills mosquitoes just before they land on hosts. An advantage of this method is that in addition to protecting participants from mosquito bites, it can be used in a standardized way in both indoor and outdoor environments. This method has shown promise as alternative to the HLC for sampling malaria vectors in Tanzania [17, 31, 32]. For instance, the first prototype achieved a sampling efficiency of ~ 60% relative to the HLC for sampling *Anopheles arabiensis* outdoors in rural Tanzania, falling to 20% when used indoors [31]. Further study on an improved prototype carried out in an urban area indicated the MET had a similar performance to the HLC [17]. A recent study evaluated a further prototype of the MET in which the electrified trapping panels were expanded to encompass the whole body of a human volunteer or calf [32], with the performance of the MET exceeding that of the HLC. The MET has not been tested yet outside Tanzania thus its effectiveness in different ecological settings is unknown. There is a need to evaluate the MET in west African settings where vector species composition, ecology and biting behaviour is often markedly different from East Africa and to see how its performance varies between sites and seasons.

This study aimed to evaluate the performance of the MET relative to the HLC in a longitudinal study in south-western Burkina Faso. Sampling was conducted over a 14-month period in 12 villages, where malaria vector abundance and species composition are known to vary considerably between seasons and sites (unpublished data). The aims were to test the performance of the MET relative to the HLC for estimating vector abundance, and location of biting (indoor vs outdoor): (i) over the study period, (ii) over the course of the night, and iii) in relation to mosquito density. Additional aims were to compare estimates of mosquito vector species composition and infection rates between HLC and MET collections and assess if they produce comparable estimates of exposure to *Anopheles gambiae* sensu lato (s.l.), based on human behaviour.

## Methods

### Study site

**Fig. 1:**
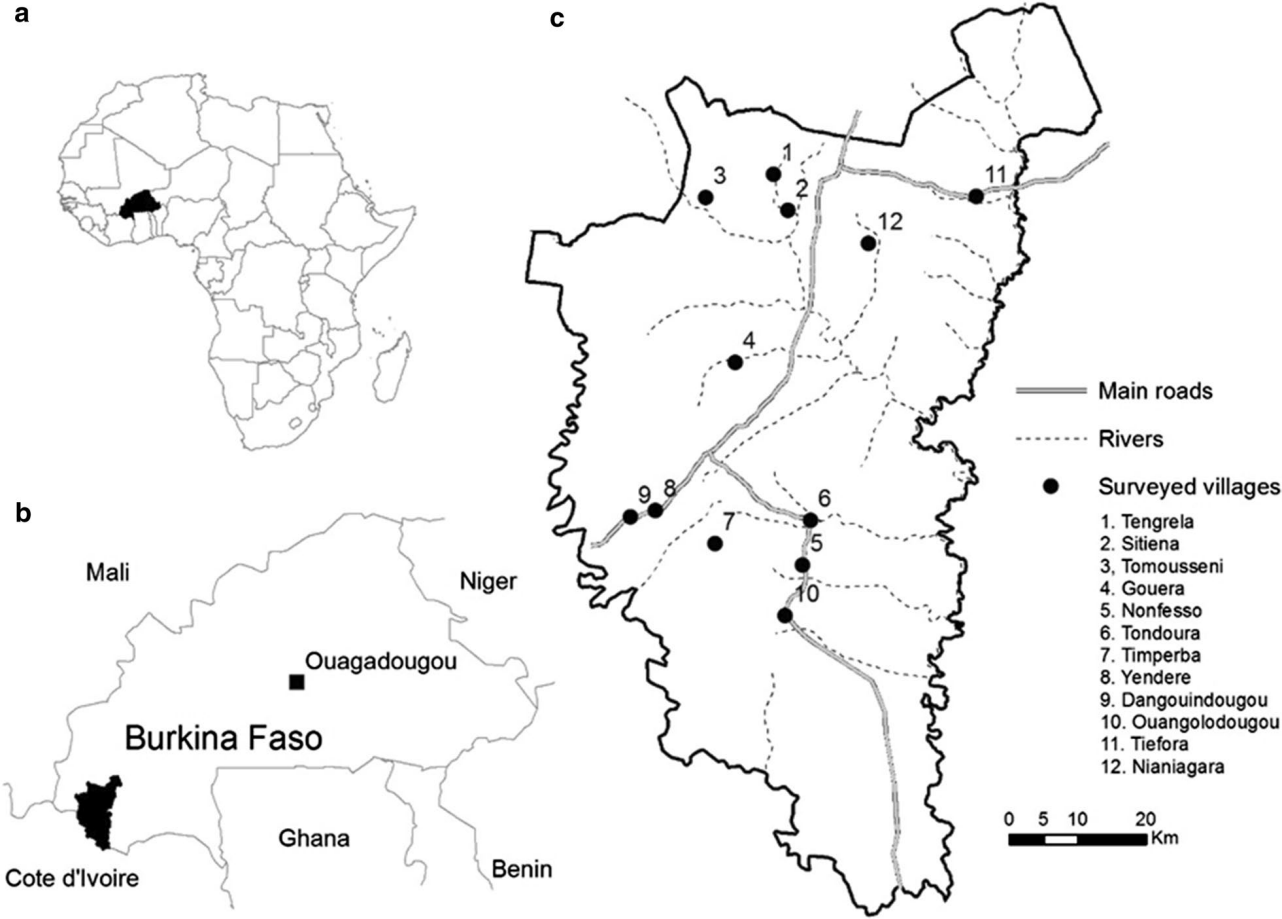
Map of the 12 study sites showing the villages for mosquito sampling. **a** Location of Burkina Faso within Africa, **b** study area in the Cascades Region, **c** villages where mosquito collection took place

This study took place in 12 villages within the Cascades Region of south-western Burkina Faso (Fig. 1), where mosquito sampling was conducted over 14 months between October 2016 and December 2017. Residents of these villages live within compounds consisting of one or more households. Most residents are subsistence farmers whose primary crops are cereals, vegetables, rice and cotton. Domestic animals including dogs, cattle, sheep, goats, pigs, donkey and poultry are usually kept within compounds. The area has two distinct seasons: a rainy season (May to October) and a dry season (from November to April) [40, 41]. Annual rainfall in the area ranges from 600 to 900 mm, with a mean temperature of 26.78 °C (range: 15.7–38.84 °C) and mean humidity of 61.89% (range: 15.11–99.95%) during the study period. *Anopheles gambiae* s.l. is the most abundant malaria (> 90%) vector in this area [42, 43].

### Trapping methods

Mosquitoes were collected using HLCs [44] and METs [31]. The MET used was an improved prototype of the version used previously [17, 31]. In brief, it consists of four 50 cm × 50 cm grid panels that can be assembled into a square with the bottom and top open. Panels are made from polyvinyl chloride (PVC) frames. Stainless steel wires (1.2 mm thick) were embedded to run from the top to bottom of each frame at a spacing of 5 mm. Adjacent wires were differentially charged as negative or positive, such that an insect would be shocked on contact with both. The assembled grid panels were connected to a power supply sourced by two 12-V batteries in series (Additional file 1: Figure S1). A protective shield made from PVC was fitted into the interior side of each panel to prevent any accidental contact between users and the electrified surface.

### Experimental design

Across the study period (Oct 2016–2017), adult mosquitoes were collected twice a month in each of the 12 villages with the occasional breaks for holidays and team training. Additionally, only one night of sampling was conducted in each village during the first month. This resulted in mosquitoes being sampled from 4 households at each village for approximately 14 months. The same group of four households was sampled on 2 nights each month; with a different group of households being selected the following month to maximize the spatial coverage of sampling within villages. There was a minimum distance of 30 m between houses sampled on the same night. This culminated in a total of 672 households being sampled over 14 months. Collections were made both inside houses and, in the peri-domestic area (within 8–10 m of the house). Indoor collections were usually conducted in the sitting rooms of houses or in single-room houses.

### Mosquito collection

On each night, host-seeking mosquitoes were collected using the HLC and MET. On the first night of sampling during each 2-day period, two houses were randomly allocated for collections with HLC and two others with METs. On the second night, these methods were rotated between households in a cross-over design. Participants involved in mosquito collections also rotated between indoor and outdoor trapping stations each hour to avoid confounding location with individual differences in attractiveness to mosquitoes.

**Fig. 2:**
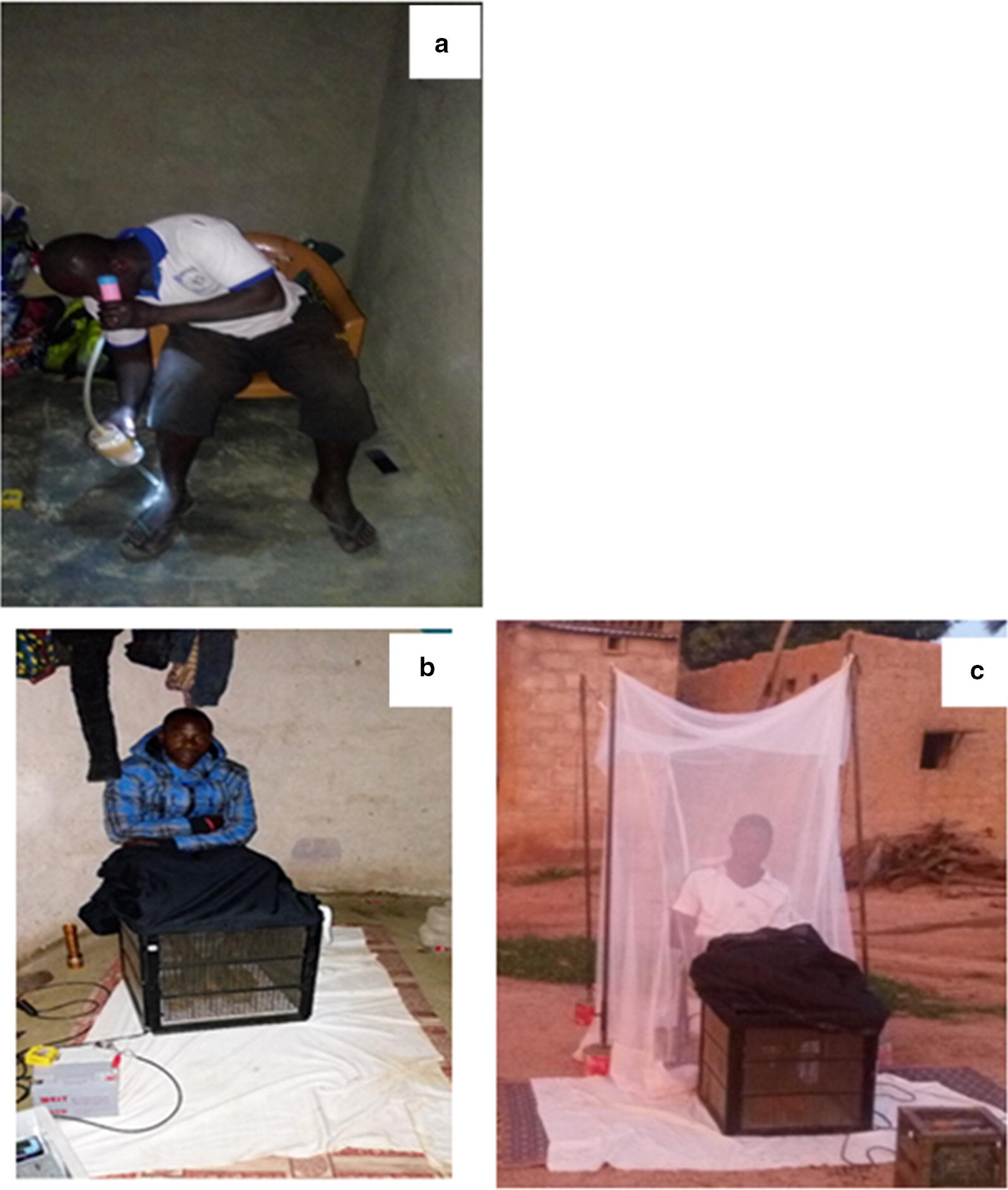
**a** A volunteer collecting mosquitoes landed on his leg using the human landing catch (HLC) method. **b**, **c** Volunteers using mosquito electrocuting traps (METs)

When collecting mosquitoes by HLC, the volunteers sat on a chair with their legs exposed up to the knees. Mosquitoes landing on their legs were sucked into pre-labelled papers cups using a mouth aspirator and a torch (Fig. 2a). For MET sampling volunteers sat on a chair with their legs up to their knees placed inside the trap (Fig. 2b, c), while the remaining part of their body was protected from mosquito bites using protective clothing (first 6 months, Fig. 2b) or a netting screen (from April 2017, Fig. 2c). The METs were placed on top of a plastic mat, which was covered with a white cloth to make it easier to see electrocuted mosquitoes that fell off the trap and onto the ground.

Each night, the HLC and MET collections were run from 7 p.m. to 6 am, with participants conducting trapping for 45 min of each hour followed by a 15-min rest break. During the break period, the MET was switched off and technicians collected mosquitoes trapped on the outer surface and those that had fallen on the white cloth using tweezers. All mosquitoes collected using METs were stored in pre-labelled Petri dishes while those collected by HLC were transferred into paper cups labelled to identify the household and trapping location (indoors or outside, trap type and collection hour).

Overall mosquitoes were sampled on 324 nights in the 14 months of data collection, culminating in a total of 1296 HLC. According to the experimental design, a similar number of HLC and MET collections should have been performed. However, due to problems with the functioning of METs and rainfall on some nights (battery problems and short circuiting) only 1080 MET collections were conducted (outdoor = 531, indoor = 549).

### Mosquito processing

Cups containing mosquitoes collected by HLC were placed into a cool box. Cotton pads soaked in a 10% sugar solution were placed on top of collection cups to feed any survivors and transferred to the laboratory. Once in the laboratory, mosquitoes were killed by putting them in a freezer, then sorted to species complex level using morphological keys [45] and stored in labelled 1.5 mL Eppendorf tubes containing silica gel. A subsample of 3199 females (36.3% of total), morphologically identified as *An. gambiae* s.l., were selected to provide a representative sample from each month, village, trapping location (indoor vs outdoor) and method (HLC, MET). The subsampling strategy was guided by consideration of the minimum sample size likely to be required to detect malaria infection in one unique mosquito collection (e.g. permutation of night, trapping method and location). Based on previous data for the study area, this was estimated as a subsample of 40 individuals. Further explanation of the rationale and strategy for this subsampling are provided in the Additional file 2: Additional information S1. Legs from individual mosquitoes from this subsample were analysed by PCR analysis to confirm their species following [46]. Likewise the head and thorax of the same specimens were tested for *Plasmodium falciparum* sporozoite infection using Enzyme-Linked Immuno-Sorbent Assay (ELISA) [47].

### Environmental data collection

During the mosquito collection, temperature (°C) and humidity (%) were recorded using Tiny Tag data loggers (Tiny Tag application Explorer 4.9) at each trapping location. Additionally, the time at which residents form the houses where the sampling is taking place go to and get out of their houses were also recorded alongside the mosquito collection.

### Statistical analysis

Analysis was conducted to test for: (i) variation in mosquito abundance between traps (per night, per hour and across the study period), (ii) density dependence in the performance of the MET relative to the HLC (iii) variation in malaria vector species composition between trapping methods (defined by the proportion of *Anopheles coluzzi* within the *An. gambiae* complex), and (iv) variation in *An. gambiae* s.l. sporozoite infection rate between traps. Additionally, (v) estimates of hourly and location-dependent (indoor vs out) produced were used to calculate and compare three key metrics of human exposure to bites generated from different trapping methods as described below [48–50]. Generalised Linear Mixed Effect Models (GLMMs) were constructed within R statistical software version 3.5.0 (2018-04-23) [51] augmented with the lme4 packages for statistical analysis [52] except for the analysis on density dependence and the variation in trap performance across the study period.

The relative efficiency of the MET compared to the HLC was assessed in terms of the number of *An. gambiae* s.l. caught per night. Mosquito abundance data were highly over-dispersed so they were modelled using a negative binomial distribution [53]. Initially, trapping method and its interaction with village and trap location were included in the maximum model of *An. gambiae* s.l. abundance along with other covariates (Model 1, Additional file 3: Table S1) to allow testing of whether trap performance varied between sites and trap location.

The variation of the relative efficiency of MET to HLC in predicting *An. gambiae* s.l. throughout the collection period was assessed separately for outdoor and indoor collection using Generalized Additive Models (GAM) with a negative binomial distribution [54]. This package allowed estimation of the nonparametric function by using a smoothing spline on week. In the full model, the response variable consists of the number of *An. gambiae* s.l. caught per night whilst the explanatory fixed effect variables were method and its interaction with the smoothing splines. To assess whether the interaction was significant in each location (indoor and outdoor), the model with interactions was compared to the basic model without interaction using the Akaike Information Criteria (AIC). Here, no random effect was included as only variation in the seasonal variation of *An. gambiae* s.l. abundance was of interest.

In addition, to test whether the relative performance of the MET compared to HLC changed over the course of night, a model was constructed with the response variable of the proportion of *An. gambiae* s.l. caught in METs in each hour of sampling out of the total in MET and HLC combined (Model 2, Additional file 3: Table S1). Here sampling “hour” was defined as a continuous variable where 1 corresponded to the first hour of collection (7 p.m. to 8 p.m.) and 11 being the last hour (5am to 6am).

Density dependence in MET performance was assessed by testing for linearity between *An. gambiae* s.l. catches in the MET and HLC following the method described in [17] using Markov Chain Monte Carlo (MCMC) in the programme Jags [55, 56]. Here the response variable was the number of *An. gambiae* s.l. collected using the MET and the explanatory variable the number collected using HLC.

Further statistical analyses relating to *P. falciparum* sporozoite rate were performed on the same subset of *An. gambiae* s.l. (n = 3199) that were individually identified to species level. In the analysis related to species composition the response variable was the proportion of *An. coluzzi* in the *An. gambiae* s.l. complex per night with explanatory variables for trapping method, location, temperature and humidity (Model 3, Additional file 3: Table S1). A similar model was constructed to analyse variation in the sporozoite rate of *An. gambiae* s.l. with the explanatory variables being mosquito species, trapping method, interaction between species and location, village, temperature and humidity (Model 4, Additional file 3: Table S1). It was not possible to include analysis of seasonality in these models because of sample sizes of mosquitoes in the dry season at some of the villages. Both data on  % *An. coluzzi* and infection rate were modelled using a binomial distribution.

Finally, data on the time and location of biting (indoors vs outside houses) were used to estimate three standard epidemiological parameters of relevance for estimating human exposure to mosquito bites and the impact of Long-Lasting Insecticide-Treated Nets (LLINs) [50, 57]. These are defined as the (i) proportion of *An. gambiae* s.l. host-seeking indoors (P_i_), (ii) proportion of mosquito bites occurring when most people are inside (time spent inside estimated based on observations, Additional file 4: Figure S2) their dwellings and likely asleep (P_fƖ_) and (iii) proportion of human exposure to *An. gambiae* s.l. bites occurring indoors π_i_). The π_i_ metric estimates the proportion of exposure to malaria transmission that occurs indoors and could be prevented using LLINs [50, 57]. These proportions were used as response variables in analyses that tested whether these exposure estimates varied between trapping methods and in response to season, temperature and humidity (Model 5–7, Additional file 3: Table S1).

In all the analysis, random effects were incorporated at the intercept to capture the baseline variability by day, compound, household and village excepted for the Model 1 (Additional file 3: Table S1). For each variable of interest, model selection was conducted through a process of backward elimination starting from a maximal model (Additional file 3: Table S1) in which likelihood ratio tests (LRTs) were used to evaluate the significance of individual terms. Mean values and 95% confidence intervals for all statistically-significant effects in the minimum model (“best model”) were obtained from the GLMMs using the effects package [58].

## Results

A total of 41,800 mosquitoes were collected over 324 trapping days, of which 41,395 were females (Additional file 5: Table S2). Most of the female mosquitoes were anophelines (86.4%), with the remainder being culicines (Additional file 5: Table S2). *Anopheles gambiae* s.l. represented 97.7% of all anophelines, (Additional file 5: Table S2). Within the subset of *An. gambiae* s.l. individually analysed to species level (n = 3199, 36.3% of total), *An. gambiae* constituted 41.58%, *An. coluzzi* 58.17% and *An. arabiensis* 0.25%. No molecular identification of species within the *Anopheles funestus* group was performed because of the small number collected indicated this is not a major vector in the area (n = 35). There was seasonal variation in vector species composition, with the proportion of *An. coluzzi* within the *An. gambiae* s.l. complex varying from ~ 75% to ~ 44% between the dry and wet season (Additional file 6: Table S3).

### Trap sampling efficiency

Overall, there were notable differences in *An. gambiae* s.l. abundance between villages, trapping methods and locations (Table 1). In addition, *An. gambiae* s.l. abundance also varied notably across the collection period, with peaks during the rainy season (May –Oct) followed by decline in the dry season (Nov-April, Additional file 7: Figure S3).

**Table 1:** Number of *An. gambiae* s.l. females collected using different trapping methods, and at different locations (indoor *versus* outdoor) across the 12 study villages between October 2016 and December 2017

Village	HLC			MET		
	Indoor	Outdoor	HLC total	Indoor	Outdoor	MET total
Dangouindougou	787	784	1571	334	454	788
Gouera	762	866	1628	113	370	483
Nianiagara	477	480	957	125	149	274
Nofesso	338	540	878	103	206	309
Ouangolodougou	268	407	675	73	82	155
Sitiena	1588	1609	3197	313	267	580
Tengrela	3407	3104	6511	1457	1323	2780
Tiefora	2276	2389	4665	1174	1125	2299
Timperba	444	414	858	225	353	578
Tondoura	550	575	1125	197	161	358
Toumousseni	787	893	1680	309	520	829
Yendere	546	676	1222	185	359	544
Total	12,230	12,737	24,967	4608	5369	9977

*HLC* human landing catch, *MET* mosquito electrocuting trap

**Fig. 3:**
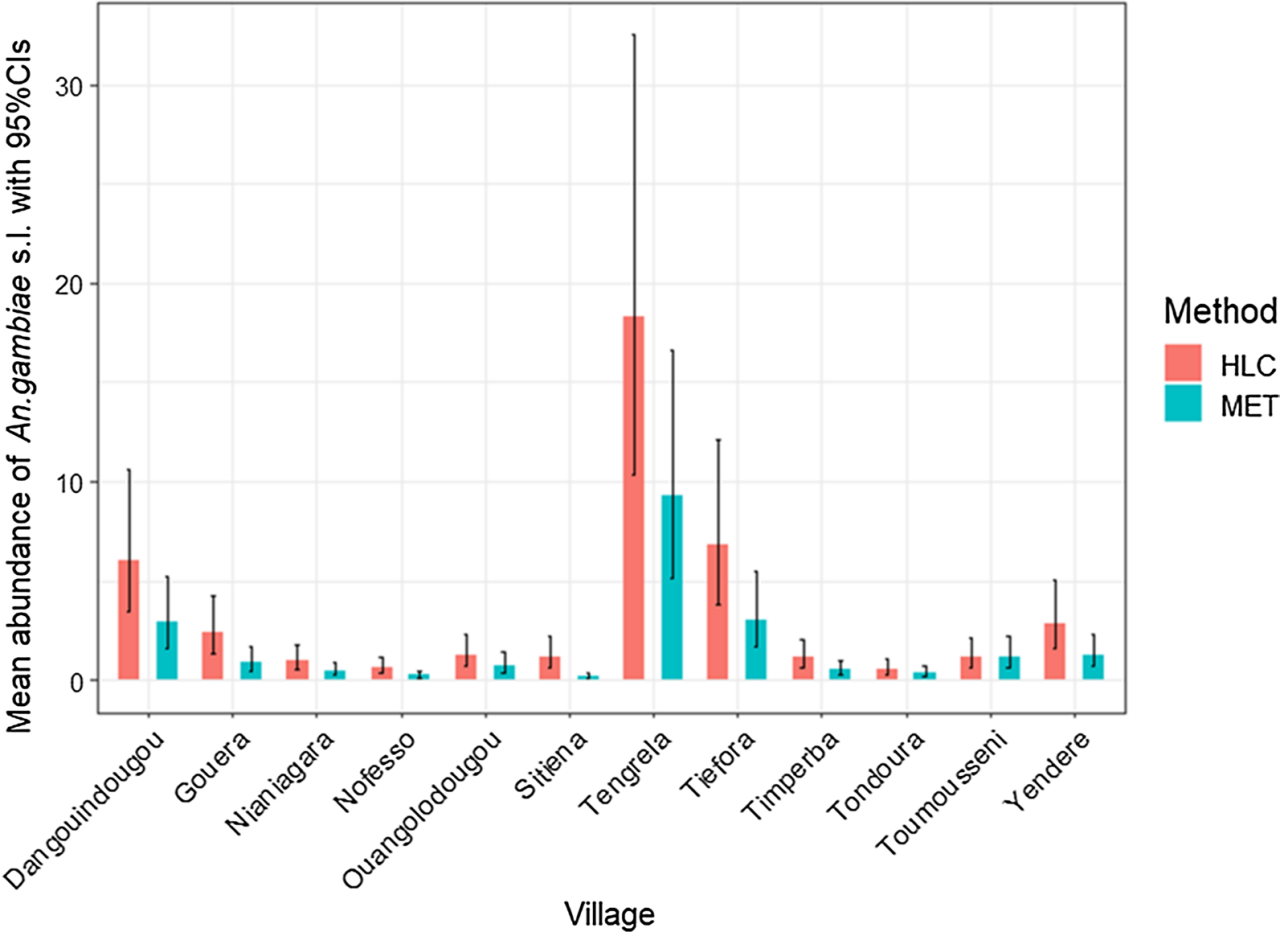
Mean predicted abundance of *An. gambiae* s.l. caught per night using different trapping methods in 12 villages in southwestern Burkina Faso. Data are pooled across trapping location (inside houses or outdoors) and the study period (October 2016 to December 2017). Error bars are with 95% confidence intervals. Here pink bars indicate HLC collection, and blue bars MET collections

**Fig. 4:**
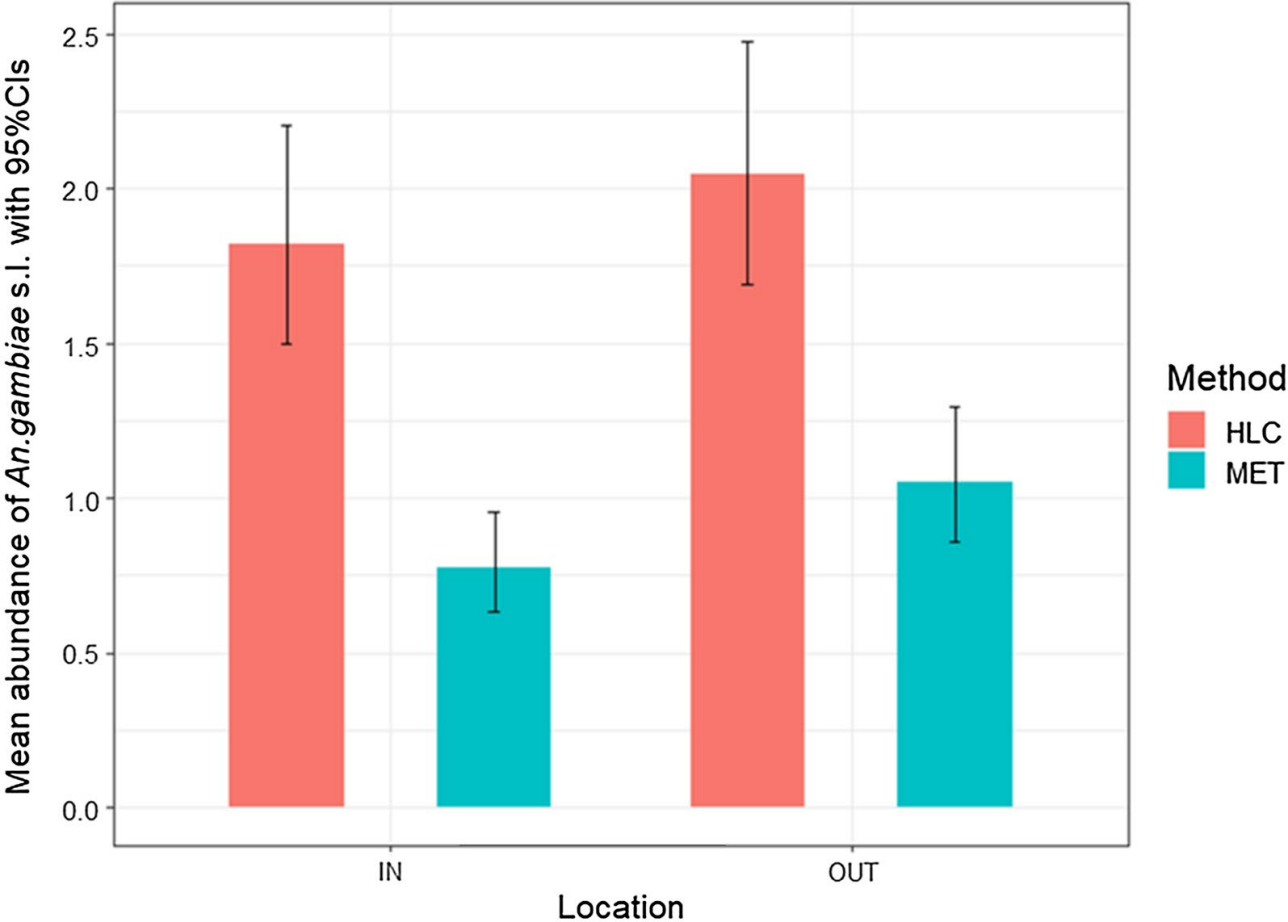
Mean predicted abundance of *An. gambiae* s.l. per night made at different trapping locations (IN = inside houses, OUT = peri-domestic area outside of houses) using two different trapping methods (pink bars = HLC; blue bars = MET) between October 2016 and December 2017. Errors bars are 95% confidence intervals

The mean abundance of *An. gambiae* s.l. was best explained in a final model that included the interaction between trapping method and village (df = 11, χ^2^ = 59.7, p < 0.0001), trapping method and location (df = 1, χ^2^ = 4.20, p = 0.04), season (as dry or wet season, (df = 1, χ^2^ = 244.42, p < 0.0001)) and humidity (df = 1, χ^2^ = 9.71, p = 0.002). The significance of these interactions indicates that there is a spatial variability in trap performance (Table 1, Fig. 3) as well as between outdoor and indoor locations (Table 1, Fig. 4). Overall the relative performance of MET compare to HLC was 46.88% (95% CI 46.20–47.42%), but there was considerable variation between villages from a low of ~ 17% relative sensitivity in Sitiena to a high of ~ 100% in Toumousseni (Fig. 3). Similarly, there was variation in trap performance between indoor and outdoor settings. However, regardless of location (in or outside), the number of *An. gambiae* s.l. collected using METs was less than the HLC (indoor: z = − 5.93, p < 0.0001; outdoor: z = − 5.42, p < 0.0001) with the performance of the MET relative to HLC being slightly higher in outdoor (Fig. 4, 51.47%;95% CI 50.89–52.22%) than indoor settings (Fig. 4, 42.86%; 95% CI 42.0–43.44%). In general, mean nightly temperatures were higher and humidity lower inside of houses than outdoors (Additional file 8: Table S4). Accounting for other significant variables in the model, *An. gambiae* s.l. abundance was positively associated with humidity (z = 3.33, p = 0.001, Additional file 9: Figure S4), and significantly higher in the wet than dry season (df = 1, χ^2^ = 244.42, p < 0.0001, Additional file 10: Figure S5), irrespective of trapping method.

### Relative performance of trapping methods across seasons

**Fig. 5:**
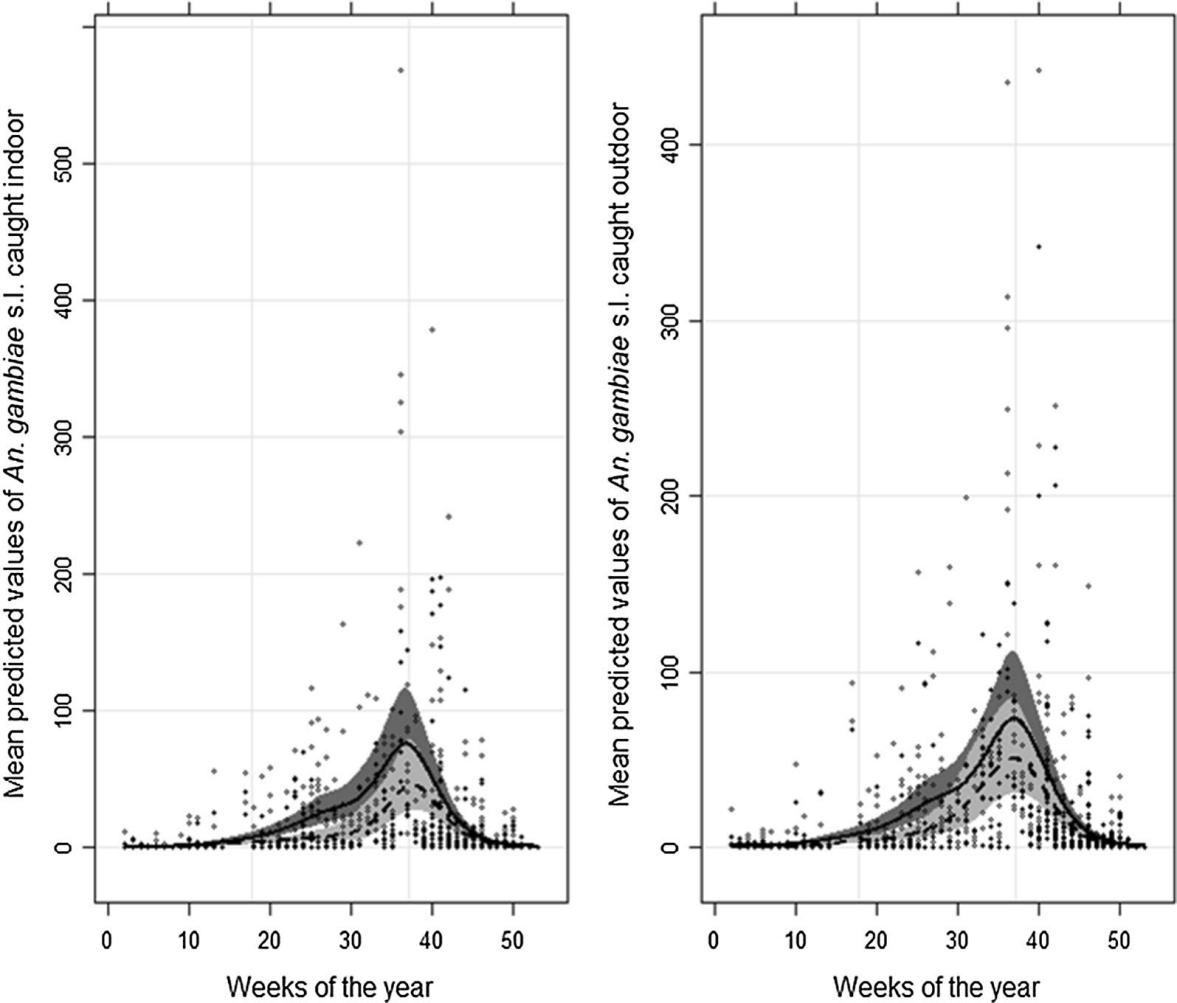
Mean predicted values of *An. gambiae* s.l. from a generalized additive model (GAM) with a negative binomial distribution. The full and open dots indicate respectively the observed number of *An. gambiae* s.l.in mosquito electrocuting trap and human landing catch through the course year indoors (left panel) and outdoors (right panel). The grey areas are the 95% confidence bands for the splines. The solid line and the dark grey indicate the data from HLC whilst the dashed-line and the light grey represents the MET. Week “1” represents the first week of January, with weeks running consecutively up to week 52 (last week of December)

Analysis by GAM indicated there was significant seasonal variation in *An. gambiae* s.l. abundance based on both indoor and outdoor collections indoors (edf = 6.697, χ^2^ = 700.3, p < 0.0001) and outdoors (edf = 6.346, χ^2^ = 624.3, p < 0.0001). However, seasonal trends in *An. gambiae* s.l. abundance were indistinguishable as predicted from MET and HLC collections. The simple model (at both indoor and outdoor) with no interaction has the lower AIC compare to model including interactions between variable method and the smoothing spline (difference in AIC are 0.55 indoor and 5.66 outdoor); indicating both methods predict similar trends (Fig. 5).

### Relative performance of trapping methods across the night

**Fig. 6:**
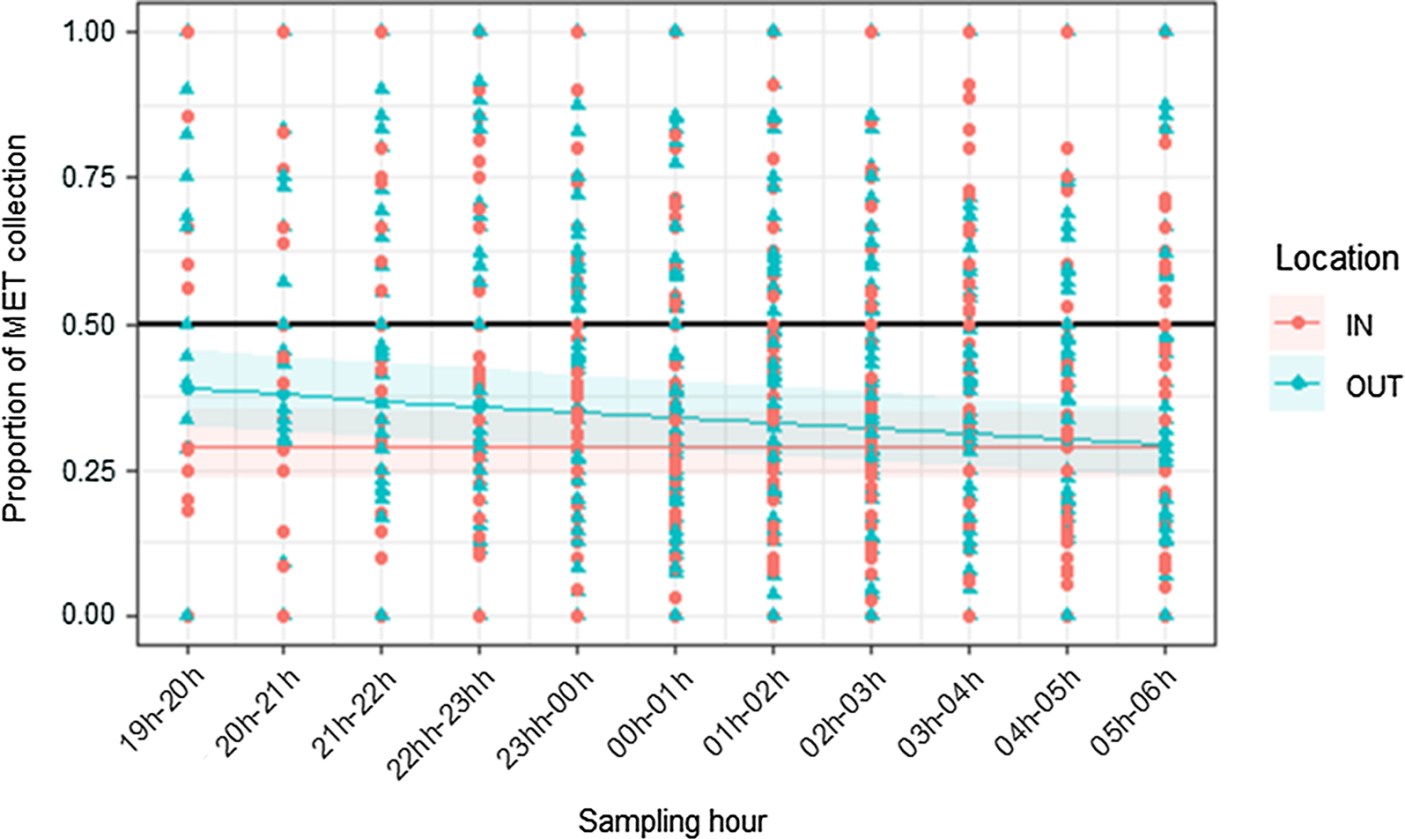
Mean proportion of *An. gambiae* s.l. caught in mosquito electrocuting trap (MET) collections relative to the human landing catch (HLC) over the course of the night (7 p.m.–6 a.m.). The red dots and blue triangles indicate the ratio MET/(MET + HLC) from the actual raw data respectively collected at indoor and outdoor sampling points. The black solid line indicates the scenario in which MET and HLC catch rates were equivalent. The red and blue lines represent the predicted regression line from models fit on data collected inside houses (IN) and outdoors (OUT). The shaded areas around the predicted lines represent 95% confidence intervals

The proportion of *An. gambiae* s.l. caught in METs relative to HLC was significantly influenced by the interaction between the sampling hour and trapping location (df = 1, χ^2^ = 10.83, p < 0.001). In indoor environments, the performance of the MET relative to the HLC stayed constant over all hours of the night (df = 1, χ^2^ = 0.13, p = 0.71). However, MET relative performance significantly declined (df = 1, χ^2^ = 27.63, p < 0.0001) between the first to the last hour of collection in outdoor settings (Fig. 6).

### The density dependence between the trapping methods

**Fig. 7:**
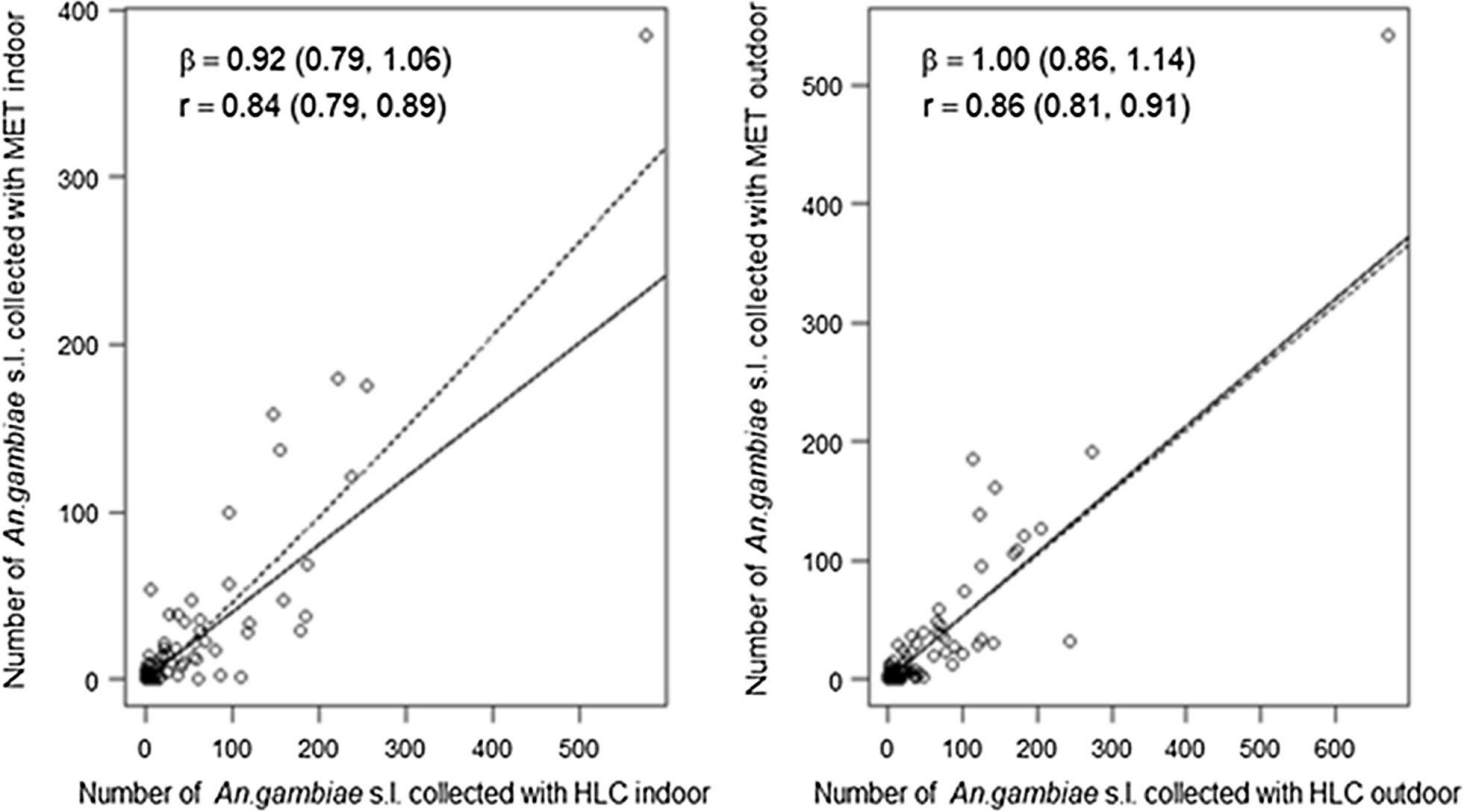
Observed values (open dots) and predicted relationships between the density of *An. gambiae* s.l. caught in mosquito electrocuting trap (MET) collections and human landing catches (HLC) at indoor and outdoor locations. In each graph, the dashed-lines indicate the model-predicted relationship between the traps and the black solid lines show the density independence relationship between MET and HLC collections

The number of mosquitoes collected using HLC ranged from 0 to 575 indoors, and 0–672 outdoors, compared to 0–385 indoors and 0–542 outdoors for the MET. The degree of dependence (β) between HLC and MET collections across this range was estimated to be 0.92 (CI 0.79–1.06) indoors and 1.00 outdoors (CI 0.68–1.14). These values indicate there was no density-dependence as the credible intervals of estimates include 1 at each location Fig. 7). There was also a strong linear correlation between the number of *An. gambiae* s.l. caught in MET and HLC collections both indoors ((r) = 0.84 (CI 0.79–0.89)) and outdoors ((r) = 0.86 (CI 0.81–91).

### Proportion of *Anopheles coluzzi* in host seeking collections

The composition of *An. gambiae* s.l. varied substantially across villages (df = 1, χ^2^ = 95.4, p < 0.0001), with *An. coluzzi* representing more than 75% of the complex at 4 villages, *An. gambiae* dominating at 6, and a roughly equal composition of *An. coluzzi* and *An. gambiae* at the remaining two sites (Additional file 11: Figure S6). The proportion of *An. coluzzi* did not vary between trapping methods (df = 1, χ^2^ = 0.027, p = 0.87), location (df = 1, χ^2^ = 0.12, p = 0.72) or in relation to the mean temperature (df = 1, χ^2^ = 2.84, p = 0.09). However, the proportion of *An. coluzzi* in collections was negatively associated with humidity (z = − 4.67, p < 0.0001; Additional file 12: Figure S7) with *An. gambiae* being more prevalent as humidity rose.

### Malaria infection

A total of 157 out of 3199 *An. gambiae* s.l. tested were positive for *P. falciparum* sporozoite infection (4.9% infection rate). Sporozoite rates varied significantly between villages (df = 11, χ^2^ = 27.63, p = 0.003), (Additional file 13: Figure S8), and in association with the interaction between vector species and trapping location (df = 1, χ^2^ = 6.15, p = 0.013). The *P. falciparum* sporozoite infection rate in *An. gambiae* was similar at indoor (5.16%; 95% CI 3.64–7.26%) and outdoor trapping locations (5.67%; 95% CI 4.17–7.66%), whereas sporozoite rates were higher in *An. coluzzi* caught indoors (5.91%; 95% CI 4.2–8.28%) than outside (2.8%; 95% CI 1.78–4.39%). However, sporozoite rates in the overall *An. gambiae* s.l. sample did not vary between trapping methods (df = 1, χ^2^ = 0.78, p = 0.38), temperature (df = 1, χ^2^ = 0.02, p = 0.88) or humidity (df = 1, χ^2^ = 0.08, p = 0.77).

### Vector behaviour and human exposure

**Fig. 8:**
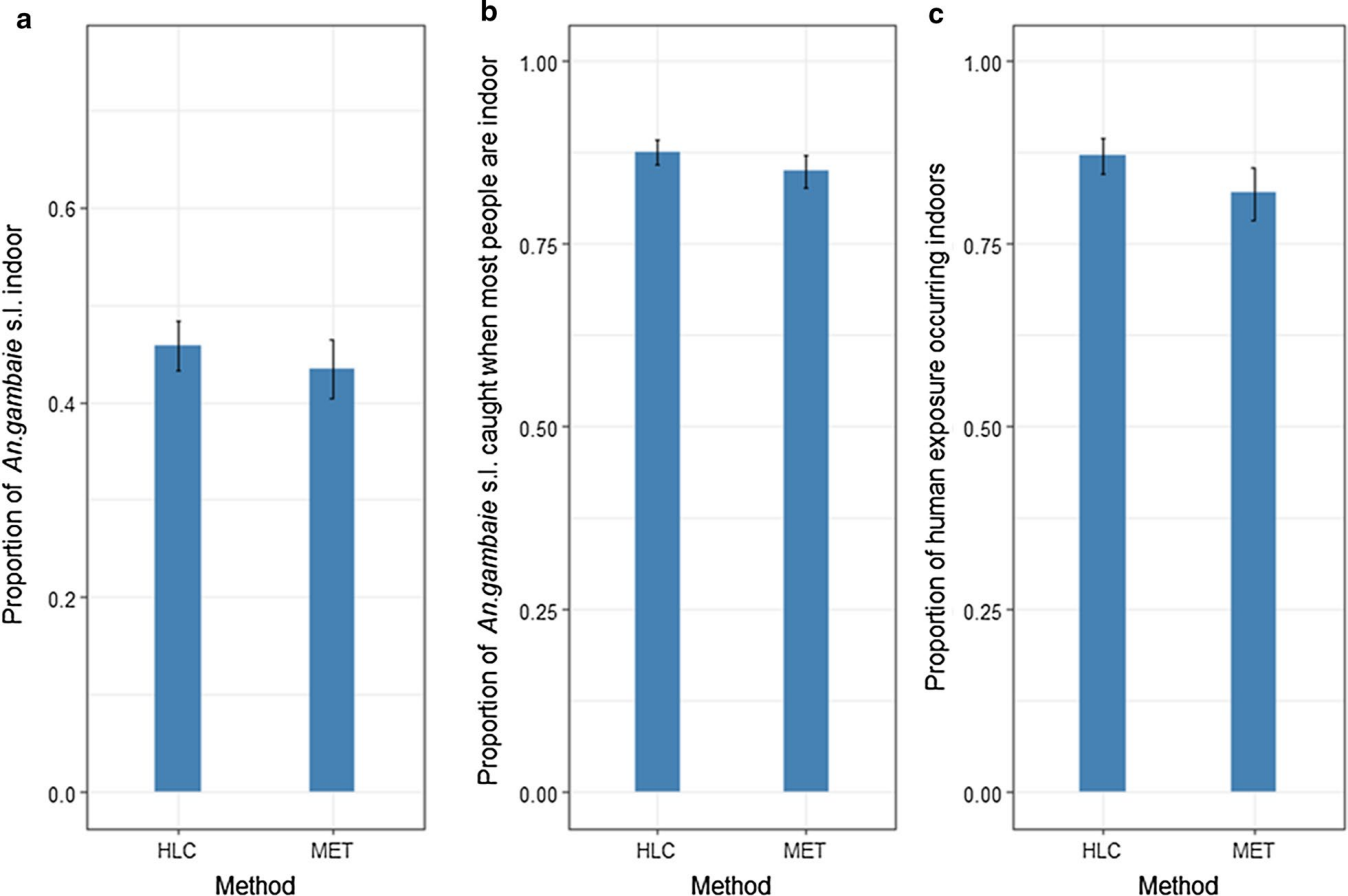
Estimates proportion of *An. gambiae* s.l. **a** caught indoor, **b** bites occurring when most people are inside their dwellings and likely asleep and **c** the proportion of human exposure to *An. gambiae* s.l. bites occurring indoors from human landing catch (HLC) and mosquito electrocuting trap (MET)

The *An. gambiae* s.l. population in the study area was relatively exophilic, with numbers host-seeking outdoors being similar or slightly higher than those indoors (Fig. 8). However, estimates of the proportion of indoor biting (P_i_) varied somewhat between trapping methods (df = 1, χ^2^ = 4.25, p = 0.039); with the HLC predicting a slightly higher degree of outdoor biting (45.73% (95% CI 43.2–48.27%) compared to the MET (43.42% (95% CI 40.47–46.4%), Fig. 8). Similarly, estimates of the proportion of *An. gambiae* s.l. caught during times when most people are indoors (P_fƖ,_ χ^2^ = 11.28, p < 0.001), and the proportion of human exposure to *An. gambiae* s.l. estimated to occur indoors (πi, χ^2^ = 21.03, p < 0.0001) were slightly but significantly higher in HLC than MET collections (Fig. 8). There was no significant additional effect of temperature, humidity or season on these human exposure traits t traits (P_i_, P_fƖ_, and πi; Additional file 14: Table S5).

## Discussion

Here the performance of the METs was evaluated as an alternative to the gold standard “HLC” for estimating human exposure to malaria vectors. This was the first time that the trap was evaluated outside Tanzania and in a West African setting. In general, the MET caught fewer *An. gambiae* s.l. than HLC with relative performance being higher in outdoor (52%) than indoor environments (43%). The overall efficiency (combining in and outdoors) of the MET (~ 46%) was similar to that described for first prototype trialled in rural Tanzania by [31], but below the near 100% relative performance reported with further prototypes tested in Tanzania [17, 32]. However, estimates of vector species composition, seasonal dynamics, biting behaviour (indoor vs outdoor) and malaria infections rates were generally similar between MET and HLC collections. This strengthens evidence that METs can provide a safe alternative to the HLC for characterizing attributes of malaria vector populations; even though they may require location-specific calibration for prediction of vector density.

It is unclear why MET performance was relatively lower in this study. However, several factors may account for this. One possibility is that the current study incorporated more intra-site variability. All previous work in Tanzania has involved evaluation at a limited number of fixed sampling points in a few sites. Here the METs were tested at multiple households across 12 different villages and noted considerable variation in MET relative performance between sites (17–100%). Thus, local characteristics of the study site may have a significant impact on trap performance. The relatively lower sampling efficiency of the MET here compared to Tanzania could also be due to operational problems that arose after the first batch of METs had been in continuous use for several months, exacerbated by wear and tear during the regular transport between villages (up to 100 km apart, on poor roads). These operational problems included short-circuits, and power supplier failure in addition to dipping in current/voltage, some of which may not have been noticed until traps failed. Although only data from days in which both MET and HLC collections were conducted was used for analysis, these faults indicate that the MET prototype may need further improvement for stable use over long periods of time. Additionally, there were small differences in trap design between the prototype used here and in Tanzania, which may have contributed to the reduced performance. For example in contrast to previous studies in Tanzania [17, 31], the MET prototype here used white non-treated net to protect the part of participant’s bodies that were not in the trap. It has been shown that *An. gambiae* s.l. are more attracted to traps with high visual contrast [22], and the use of white netting to protect participants here may have diminished the contrast between the trap and host bait compared to previous versions. Another factor that can make difference is the vector ecology and species composition. The major vectors in areas where the MET has been used in Tanzania is *An. arabiensis* [17, 31] whereas *An. gambiae* and *An. coluzzi* were the main vectors in our study area in Burkina Faso [42, 43]. Cuticular hydrocarbon composition (CHC) varies between Anopheles species [59–61], and it is known that the electrical conductivity of insects can vary with their CHC, water content and body size [62]. Therefore, the variation in the MET performance between the current study and those carried out in Tanzania could also be due to local variation in vector species composition.

The results from the present study suggested METs performed better in outdoor ~ 52% relative sensitivity compared to the HLC) than indoor (~ 43%) settings. Earlier trials in Tanzania also found MET performance to be higher outdoors than inside houses [31]. It is unclear why MET sampling efficiency tends to be higher outdoors, with further work required to address this bias. Given the growing recognition of the importance of outdoor biting in maintaining residual malaria transmission [28–30] and current lack of satisfactory alternatives to the HLC for measuring this, the MET can serve a useful purpose even if only suitable for use outdoors. The relatively good performance of the MET relative to the HLC for sampling malaria vectors outdoors reported here and elsewhere [17, 32] indicate that it is suitable for monitoring exophagic and zoophilic vector [32] populations.

The relative efficiency of the MET for collection of *An. gambiae* s.l. across dry and wet seasons was evaluated, and its ability to reflect seasonality in vector abundance relative to the HLC standard. Both trapping methods confirm strong temporal variability in vector abundance, likely due to seasonality and meteorological conditions as has been widely documented in Burkina Faso and other parts of West Africa [63, 64]. The current results indicate that the relative performance of the MET compared to the HLC stays constants across seasons, and that both methods predict similar seasonal trend in vector abundance. Additionally, there was no evidence of density dependence in the sampling efficiency of METs over a wide range of *An. gambiae* s.l. density. This contrasts with results from an earlier prototype where MET performance showed signs of density dependence indoors but not outside [17], but another study also found no density dependence [31]. However, this previous study was conducted over a relatively short period (21 nights) and did not encapsulate the seasonal extremes in vector density incorporated here. Based on the current and previous studies, it can be concluded that the MET can provide relatively accurate estimates of vector population dynamics that are unbiased by season or underlying density. An investigated was also undertaken to assess whether the performance of the MET relative to the HLC decreased over the course of a sampling night as could be indicative of battery drain. Consistent with previous studies [17, 31], there was no detection of any difference in MET sampling efficiency throughout the night when it was used indoors. However, there was a reduction in relative MET performance throughout the night when used outdoors. Such a decrease in MET sampling efficiency outdoors was reported with an early MET prototype in Tanzania [31], but not in a follow up with a new version [17]. It is unclear why MET sampling efficiency falls during the night in outdoor but not indoor settings. One possibility is variation in microclimatic conditions like humidity, which is generally higher outdoors than indoors. Humidity can trigger more rapid discharge of batteries [65]. To maintain consistent MET performance when used outdoors, batteries could be changed during the sampling night.

The malaria vector species composition in this study area varied notably compared to that of previous MET trials in Tanzania. Specifically *An. coluzzi* and *An. gambiae* were the dominant vector species here compared to *An. arabiensis* and *An. funestus* in Tanzania [17, 32, 66, 67]. Previous work in Tanzania indicated MET capture efficiency varied between malaria vector species (e.g. *An. arabiensis* and *An. funestus* [31]). However, vector species composition was similar in collections made by HLC and MET here; indicating no differential sampling performance between *An. coluzzi* and *An. gambiae*. Further calibration may be required to ensure the MET gives unbiased estimates of composition of malaria vector species in new settings. Similar to previous studies [17, 32], we found no difference in malaria sporozoite rates between vectors in HLC and MET collections. Thus, the MET also appears to yield unbiased estimates of appropriate for estimating of *An. gambiae* s.l. infection rates and transmission potential.

Finally, Three key human-mosquito exposure metrics were evaluated to assess whether they were reliably predicted by the MET: the proportion of (i) indoor biting (P_i_), (ii) *An. gambiae* s.l. bites occurring during times when most people are indoors (P_fƖ,_) and (iii) human exposure to *An. gambiae* s.l. bites that would occur indoors in the absence of personal or household physical protection (π_i)_ [50]. A higher proportion of outdoor biting by *An. gambiae* s.l. was found than previously reportedly in Burkina Faso [68–70]. In general, estimates of these three exposure-metrics were similar between HLC and MET collections. However, the MET tended to slightly underestimate all three metrics likely because of its slightly lower sampling performance in indoor *versus* outdoor settings. However even this with bias estimates of exposure as calculated by the different trapping methods were generally within a few percentage points of one another. For operational use, estimates of exposure derived from MET collections could be adjusted to compensate for this bias.

The multi-site nature of this study allowed assessment of wider aspects of MET feasibility for programmatic sampling. In contrast to previous trials in Tanzania where the MET was used in fixed, single locations [17, 31]; here was carried out in 12 villages requiring the MET to be moved every few days and sometimes as far as 100 km. The integrity of electrified surfaces on the METS were checked before and after transport in the field. The output voltage was also regularly checked during collections to ensure it was meeting the necessary target. On occasions where voltage output was suboptimal (~ 0.4% of days), MET operation was stopped and the problem reported to technical support team. Overall, MET collections were performed on ~ 17% fewer sampling hours than the HLC. However, this does not represent the proportion of times that the MET failed. Most of these MET hours (~ 9%) were lost while waiting for a replacement unit to be made and delivered (~ 4-week period). The most frequent problem encountered with MET use was power failure due to short-circuiting (~ 6% of time) with occasional sparking on the frame. Therefore, further improvements in MET design are needed to resolve this issue. In addition, it was noted that short-circuiting was more likely to occur when there was high level of moisture in the environment (e.g. rainy season, times of high humidity). This was probably due to small water droplets condensing on the frame and occasionally running down the wires. Regular wiping of the MET surface (e.g. during 15 min break periods from sampling) could help avoid a build-up moisture of trap surface. Alternately, redesigning the trap with wires running horizontally instead of vertically will prevent droplets from running down into the frame. METs were subjected to heavy use in this study, under challenging field conditions. It is perhaps not surprising that traps exhibited some degree of physical damage and breakage under these intense circumstances. These issues could be resolved by making future prototypes more robust, and/or keeping METs in fixed locations rather than in constant transport. In addition, on some other nights, MET sampling was intentionally stopped (~ 1% of the sampling hours) due to high wind and rainfall that was anticipated to drive water onto the MET surface and cause short-circuiting. Even with these difficulties, the METs still performed relatively well and consistently with the HLC in this study. To increase the protection of volunteers from bites of very small biting insects (those with wingspan less than 5 mm) that may be present at some study sites, we recommend fitting fine-mesh insecticide-free netting on the inner panel of MET surfaces with very small holes.

An additional consideration is the relative expense of doing collections with METs *versus* HLC. Currently, MET are individually built to order by a small team; with the combined cost for all components and manufacture of ~ £ 650–700 per unit. This cost is prohibitively high for large-scale surveillance (e.g. by comparison, a standard CDC light trap costs ~ $ 100 USD per unit). However, it is anticipated that the production cost would significantly decrease if produced at scale. While costs of MET collections may always be more expensive than a simple HLC where no equipment is required, we believe this additional expenditure is justified in terms of the improved safety to human subjects that it can provide.

## Conclusions

This is the first-time that the MET was evaluated outside of East Africa. Overall, the MET collected proportionately fewer malaria vectors than the HLC, and slightly overestimated the proportion of outdoor biting. However, the performance of METs relative to the HLC was consistent over time, and provided similar estimates of seasonal dynamics, biting behaviour, species composition and infection rates in malaria vector populations. Thus, despite some technical problems arising after prolonged MET usage under field conditions, we conclude it presents a promising and safer alternative for monitoring human exposure to malaria vectors in outdoor environments.

## Supplementary information

**Additional file 1.** Assembled Mosquito Electrocuting trap used for mosquito collections, connected to the power supplier and the 12-volt batteries.

**Additional file 2.** Subsampling strategy.

**Additional file 3.** Maximal models used for the modelling including the primary response variable, explanatory variables and statistical distribution used.

**Additional file 4.** Graphs indicating the proportion of residents (at households where mosquitoes were being collected) that were observed to be inside their houses during different hours of the night.

**Additional file 5.** Number of mosquitoes collected pooled over the collection methods (Human landing catch and mosquito electrocuting trap) and displayed by species and per village over 15 months (October 2016 to December 2017). Totals include both female and male mosquitoes.

**Additional file 6.** Number of *An. gambiae* complex females that tested positive for the presence of *P. falciparum* sporozoites during dry (November 2016 to March 2017 and November to December 2017) and wet season (October 2016) with different trapping methods (HLC, MET) and at different trapping locations (indoors versus outside of houses). Numbers represent totals pooled over study villages and the collection period (October 2016 to March 2017).

**Additional file 7.** Number (raw data) of *An. gambiae s.l.* collected per month from (October 2016 to December 2017 by trapping methods **a** indoor and **b** outdoor using mosquito electrocuting trap (MET) and human landing catch (HLC).

**Additional file 8.** Range of average temperature (°C) and relative humidity (%) recorded at the mosquito collection point using data logger.

**Additional file 9.** Effect of the humidity on the mean predicted number of *An. gambiae s.l.* collected per night according over the trapping methods, location and village. The solid black line indicates the regression line based on the model and grey-shaded area indicates the 95% CIs. Humidity data were only available for part of the sampling period (e.g mostly during the dry season months [Nov 2016 to April 2017, and Nov to Dec 2017], and a few months in wet season [October 2016 and May to October 2017]. The predicted relationship between relative humidity and vector abundance is thus based on months in which matched data were available.

**Additional file 10.** Mean predicted number of *An. gambiae s.l.* collected per night and season over the trapping methods, location and village with 95% CIs. Dry season indicates *An. gambiae s.l.* collected from November to April whilst wet season corresponds to period between May and October.

**Additional file 11.** Mean predicted proportion of *An. coluzzii* relative to *An. gambiae* collected per village from October 2016 to December 2017, pooled over the trapping location and methods, with 95% CIs.

**Additional file 12.** Effect of the mean relative humidity on the estimation of the proportion of *An. coluzzii*. The solid black line is the regression line of the predicted proportions and the grey-shaded area indicate the 95% CIs.

**Additional file 13.** Mean predicted *Plasmodium falciparum* infection rate in *An. gambiae s.l.* collected per village from October 2016 to December 2017, pooled over the trapping location and methods, with 95% CIs.

**Additional file 14.** Non-significant term excluded from the best models where LRT represents likelihood ratio test and degree of freedom of 1 for all the terms.

## Data Availability

Material & Data Transfer Agreements (MDTA) will be used when sharing data between members of the consortium who did not generate original data. Public release of data will be timed to follow publication. Earlier use for access to the data will be considered according to the conditions of the National/Institutional Ethics Review Boards. Data can be made available after a written expression of request for data with no apparent competing interest and in compliance with the “MIRA project”, the Centre National de Recherche et de Formation sur le Paludisme (CNRFP) and University of Glasgow data sharing agreements.

## Abbreviations

AIC: Akaike Information Criterion
CDC-LT: Centre Disease Control Light Trap
EIR: entomological inoculation rate
HDN: human-baited double net
HDT: host decoy trap
ELISA: enzyme linkage immuno-sorbent assay
GAM: generalized additive models
GLMM: generalized linear mixed models
HLC: human landing catches
ITT-C: Ifakara tent trap design C
LLINs: long-lasting insecticide-treated nets
LRT: likelihood ratio tests
MCMC: Markov chain Monte Carlo
MET: mosquito electrocuting trap
PCR: polymerase chain reaction
PVC: polyvinyl chloride
