# Supplementary material for: Evaluation of mosquito electrocuting traps as a safe alternative to the human landing catch for measuring human exposure to malaria vectors in Burkina Faso
Source: Malar J. 2019 Dec 2;18:386. doi: 10.1186/s12936-019-3030-5 (PMC6889701; doi:10.1186/s12936-019-3030-5)
Supplement: Supplementary file 1 — Additional file 1. Assembled Mosquito Electrocuting trap used for mosquito collections, connected to the power supplier and the 12-volt batteries. [file 12936_2019_3030_MOESM1_ESM.pptx]

## Slide 1
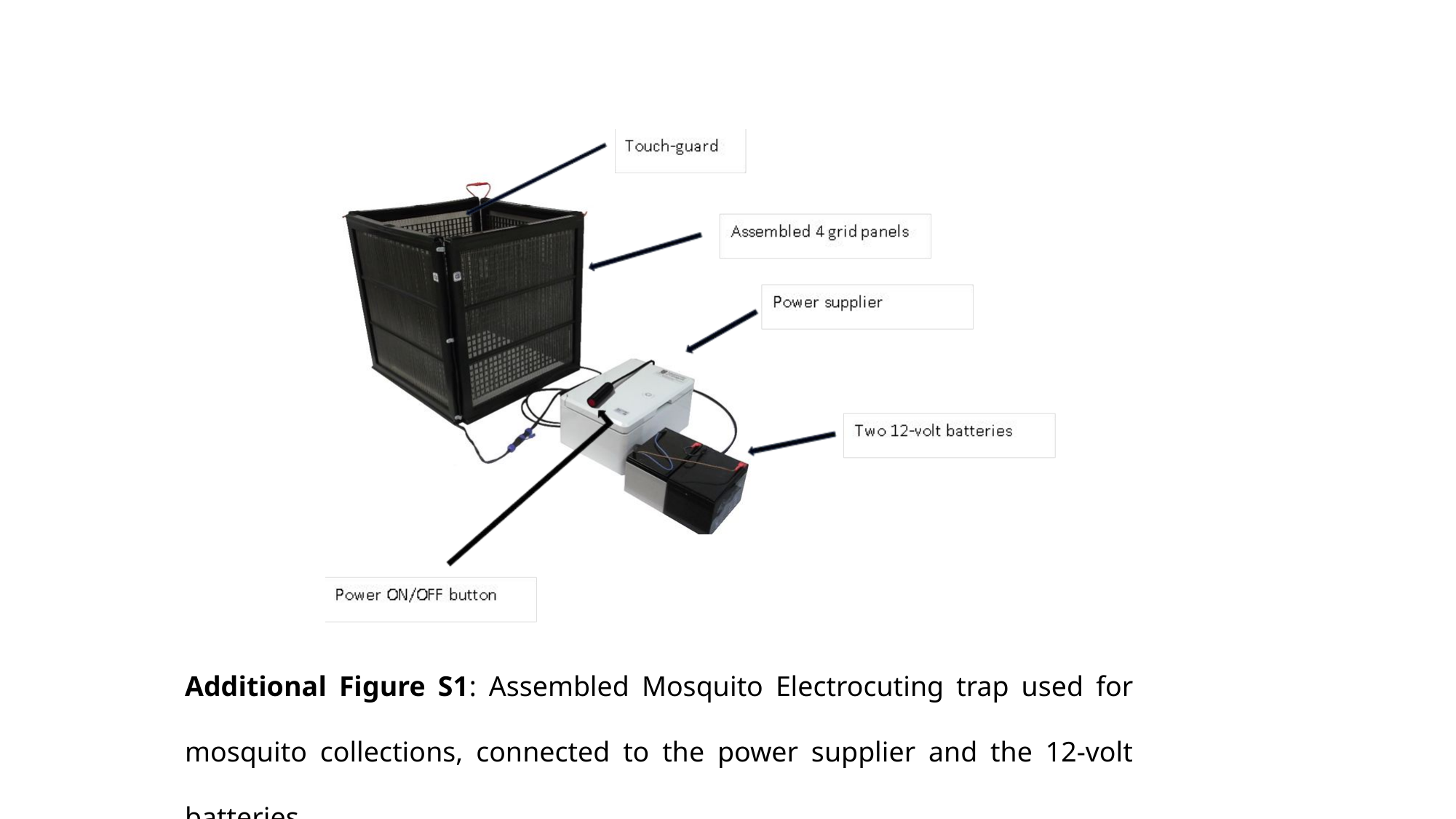

Additional Figure S1: Assembled Mosquito Electrocuting trap used for mosquito collections, connected to the power supplier and the 12-volt batteries.
