# Supplementary material for: Evaluation of mosquito electrocuting traps as a safe alternative to the human landing catch for measuring human exposure to malaria vectors in Burkina Faso
Source: Malar J. 2019 Dec 2;18:386. doi: 10.1186/s12936-019-3030-5 (PMC6889701; doi:10.1186/s12936-019-3030-5)
Supplement: Supplementary file 2 — Additional file 2. Subsampling strategy. [file 12936_2019_3030_MOESM2_ESM.docx]

**Additional Information S1:** Based on baseline data collected in nearby areas [[35](#_ENREF_35)], the average the *Plasmodium falciparum* sporozoite infection rate in *An. gambiae s.l.* was estimated as ~5% [[35](#_ENREF_35)]. Assuming rates are similar in our study area, we would need to test a minimum of 40 females from each group of interest to have a chance of detecting two infected individuals. On this basis, we proposed to subsample ~40 female *An. gambiae* s.l. from each trapping method (HLC and MET) and location (indoors and outdoors) on each night of sampling both for testing for *P. falciparum*. and for carrying PCR analysis to assess the proportion of *An. coluzzii in* the *An. gambiae* s.l. complex. By aiming to analyze a roughly similar number of individuals, the relative precision with which these two proportions could be estimated was standardized across collections. It was possible to achieve this sample size in the rainy season of 2016 (October) and 2017 (June to October) when mosquito densities were high, but not always during the dry season (November 2016 to May 2017) when densities were much lower. Consequently, all sample were subjected to molecular analysis when this number was lower than forty (the mean number analyzed per collection in these dry months was ~13).
