# Supplementary material for: Evaluation of mosquito electrocuting traps as a safe alternative to the human landing catch for measuring human exposure to malaria vectors in Burkina Faso
Source: Malar J. 2019 Dec 2;18:386. doi: 10.1186/s12936-019-3030-5 (PMC6889701; doi:10.1186/s12936-019-3030-5)
Supplement: Supplementary file 3 — Additional file 3. Maximal models used for the modelling including the primary response variable, explanatory variables and statistical distribution used. [file 12936_2019_3030_MOESM3_ESM.docx]

**Additional Table S1:** Maximal models used for the modelling including the primary response variable, explanatory variables and statistical distribution used.

| **Model** | **Tests** | **Response variables** | **Fixed Effect variables** | **Random effect variables** | **Type of data** | **Distribution** |
| --- | --- | --- | --- | --- | --- | --- |
| 1 | Trap efficiency | Abundance | Village+ Method + Location + Season  Village: Method + Location: Method + Season: Method + Temperature+ Humidity, | Date + Compound + Household | *Nightly An. gambiae* s.l. collection data | Negative binomial in GLMMs |
| 2 | Proportion of MET collection across night | MET_ total/ (MET_ total + HLC_ Total) | nHour + Location + nHour: Location, | Date + Village | Hourly *An. gambiae* s.l. collection data | Binomial in GLMMs |
| 3 | Proportion of *An. coluzzii* | (An. coluzzii/An. coluzzii + An. gambiae) | Village + Method + Location  + Temperature+ Humidity | Date + Compound + Household | Subset of *An. gambiae* s.l. data | Binomial in GLMMs |
| 4 | Sporozoite infection rate | Positive/ (Positive + Negative) | Village + Method + Location  + Location: Species + Temperature+ Humidity, | Date + Compound + Household | Subset of *An. gambiae* s.l. data | Binomial in GLMMs |
| 5 | Proportion of indoor biting (P_i_) | I_7pm -> 6am /_ (I_7pm -> 6am_ + O_7pm -> 6am_) | Method + Season + Season: Method + Temperature + Humidity, | Village + Date + Compound + Household | *An. gambiae* s.l. data | Binomial in GLMMs |
| 6 | Proportion of mosquito when people are indoor (P_fƖ_) | (I_10pm->5am +_ O_10pm->5am_) / (I_7pm->6am_ + O_7pm->6am_) | Method + Season + Season: Method+ Temperature + Humidity, | Village + Date + Compound + Household | *An. gambiae* s.l. data | Binomial in GLMMs |
| 7 | Human exposure to mosquito bite indoor (π_i_) | I_10pm -> 5am_ / (I_10pm -> 5am_ + O_7pm -> 10pm, 5am -> 6am_) | Method + Season + Season: Method + Temperature + Humidity, | Village + Date + Compound + Household | *An. gambiae* s.l. data | Binomial in GLMMs |

Methods are MET: Mosquito Electrocuting trap and HLC: Human Landing Catch and location indicates indoors versus outdoors. The average temperature and relative humidity were obtained by averaging the records over the course of the collection night. Here locations are the collection points inside houses or outdoor while seasons are dry or wet seasons. nHour represents here hours as discrete variables from 1 being the first hour of collection (7pm-8pm) to the last hour of collection of the night being 11 (5am – 6am). nMonth describes month as discrete variables from the first month (October 2016) of collection to the last month of collection (December 2017). The season was defined here as categorical variable dry (November to April) or wet (May to October). P_i_ was calculated as the number of *An. gambiae* sl. caught indoors (I) divided by the total caught indoors (I) and outdoors (O) over a sampling night (7pm- 6am): I_7pm -> 6am_ / (I_7pm -> 6am_ + O_7pm -> 6am_) (Govella et al., 2010, Russell et al., 2011). P_fl_ is the number of *An. gambiae* s.l. collected during hours when more than 50% of people are indoors and could be protected by LLINs, divided by the total caught over the entire night of sampling (in and out). This P_fl_ was calculated by dividing the total *An. gambiae* s.l. collected between 10pm and 5am indoors and outdoors (I_10pm->5am +_ O_10pm->5am_) by the total collected between 7pm and 6am (I_7pm->6am_ + O_7pm->6am_) (Govella et al., 2010, Russell et al., 2011). Values of π_i_ were computed as the proportion of total *An. gambiae* s.l. collected indoors during hours when people could be protected by an LLIN (I_10pm -> 5am_) over itself and the total *An. gambiae* s.l. collected outside during non-sleeping hours (I_10pm -> 5am_ + O_7pm -> 10pm, 5am -> 6am_) (Govella et al., 2010). Here, “subset of *An. gambiae* s.l. “refers to subset that were individually identified to species levels and individually tested for sporozoite infection.
