# Supplementary material for: Evaluation of mosquito electrocuting traps as a safe alternative to the human landing catch for measuring human exposure to malaria vectors in Burkina Faso
Source: Malar J. 2019 Dec 2;18:386. doi: 10.1186/s12936-019-3030-5 (PMC6889701; doi:10.1186/s12936-019-3030-5)
Supplement: Supplementary file 4 — Additional file 4. Graphs indicating the proportion of residents (at households where mosquitoes were being collected) that were observed to be inside their houses during different hours of the night. [file 12936_2019_3030_MOESM4_ESM.pptx]

## Slide 1
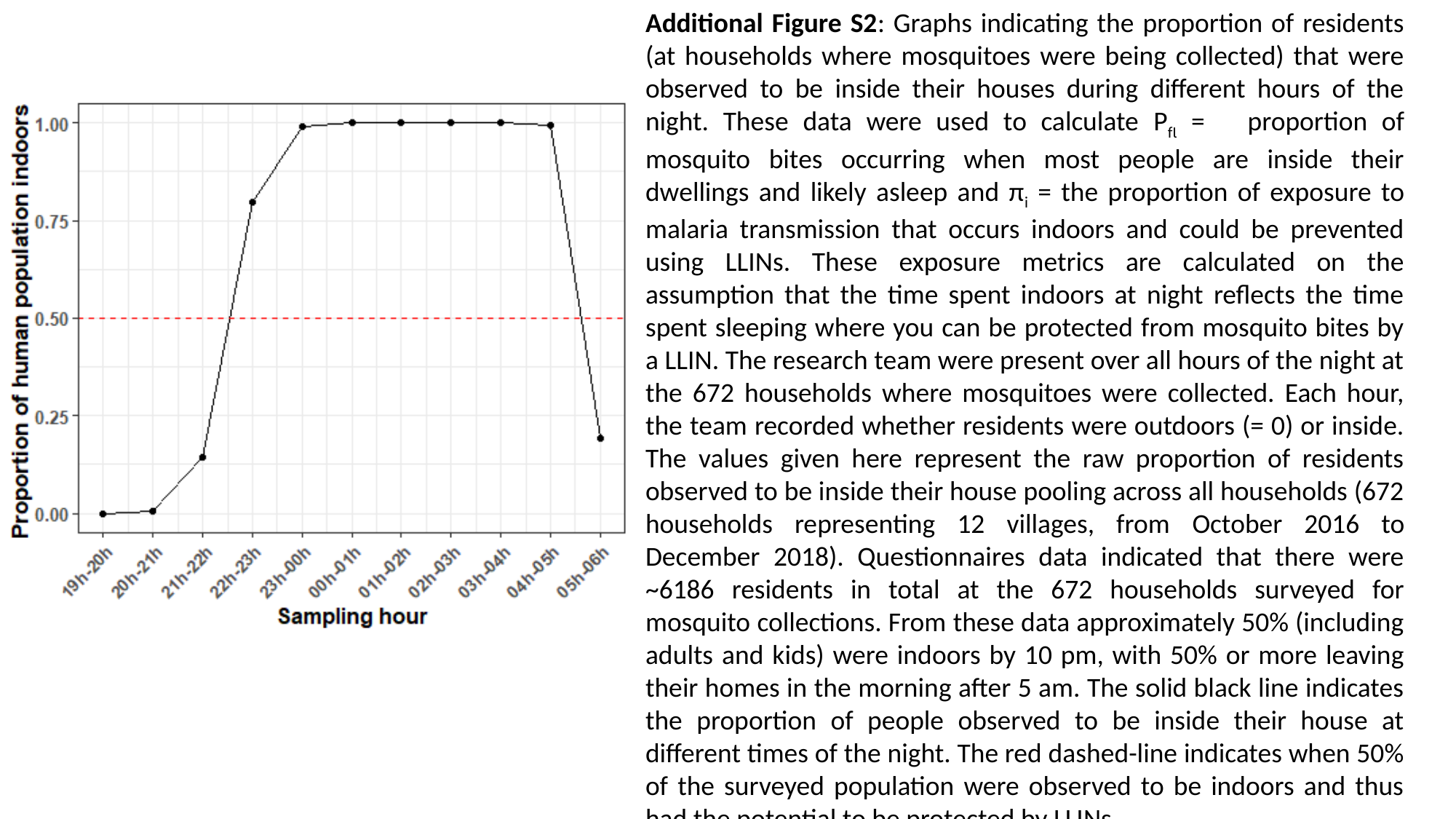

Additional Figure S2: Graphs indicating the proportion of residents (at households where mosquitoes were being collected) that were observed to be inside their houses during different hours of the night. These data were used to calculate PfƖ = proportion of mosquito bites occurring when most people are inside their dwellings and likely asleep and πi = the proportion of exposure to malaria transmission that occurs indoors and could be prevented using LLINs. These exposure metrics are calculated on the assumption that the time spent indoors at night reflects the time spent sleeping where you can be protected from mosquito bites by a LLIN. The research team were present over all hours of the night at the 672 households where mosquitoes were collected. Each hour, the team recorded whether residents were outdoors (= 0) or inside. The values given here represent the raw proportion of residents observed to be inside their house pooling across all households (672 households representing 12 villages, from October 2016 to December 2018). Questionnaires data indicated that there were ~6186 residents in total at the 672 households surveyed for mosquito collections. From these data approximately 50% (including adults and kids) were indoors by 10 pm, with 50% or more leaving their homes in the morning after 5 am. The solid black line indicates the proportion of people observed to be inside their house at different times of the night. The red dashed-line indicates when 50% of the surveyed population were observed to be indoors and thus had the potential to be protected by LLINs.
