# Supplementary material for: Evaluation of mosquito electrocuting traps as a safe alternative to the human landing catch for measuring human exposure to malaria vectors in Burkina Faso
Source: Malar J. 2019 Dec 2;18:386. doi: 10.1186/s12936-019-3030-5 (PMC6889701; doi:10.1186/s12936-019-3030-5)
Supplement: Supplementary file 5 — Additional file 5. Number of mosquitoes collected pooled over the collection methods (Human landing catch and mosquito electrocuting trap) and displayed by species and per village over 15 months (October 2016 to December 2017). Totals include both female and male mosquitoes. [file 12936_2019_3030_MOESM5_ESM.docx]

**Additional Table S2**: Number of mosquitoes collected pooled over the collection methods (Human Landing Catch and Mosquito Electrocuting Trap) and displayed by species and per village over 15 months (October 2016 to December 2017). Totals include both female and male mosquitoes.

| Village | *Culex sp* | *Mansonia sp* | *Aedes sp* | *Anopheles sp* | *An. gambiae sl* |
| --- | --- | --- | --- | --- | --- |
| Dangouindougou | 1118 | 1494 | 21 | 2411 | 2359 |
| Gouera | 277 | 80 | 18 | 2131 | 2111 |
| Nianiagara | 10 | 30 | 20 | 1252 | 1231 |
| Nofesso | 9 | 8 | 3 | 1188 | 1187 |
| Ouangolodougou | 16 | 9 | 11 | 833 | 830 |
| Sitiena | 53 | 186 | 8 | 4015 | 3777 |
| Tengrela | 260 | 1239 | 7 | 9540 | 9291 |
| Tiefora | 52 | 147 | 7 | 7185 | 6964 |
| Timperba | 105 | 35 | 43 | 1449 | 1436 |
| Tondoura | 2 | 19 | 17 | 1491 | 1483 |
| Toumousseni | 106 | 179 | 11 | 2566 | 2509 |
| Yendere | 96 | 237 | 6 | 1800 | 1766 |
| Total | 2104 | 3663 | 172 | 35861 | 34944 |
