# Supplementary material for: Evaluation of mosquito electrocuting traps as a safe alternative to the human landing catch for measuring human exposure to malaria vectors in Burkina Faso
Source: Malar J. 2019 Dec 2;18:386. doi: 10.1186/s12936-019-3030-5 (PMC6889701; doi:10.1186/s12936-019-3030-5)
Supplement: Supplementary file 6 — Additional file 6. Number of An. gambiae complex females that tested positive for the presence of P. falciparum sporozoites during dry (November 2016 to March 2017 and November to December 2017) and wet season (October 2016) with different trapping methods (HLC, MET) and at different trapping locations (indoors versus outside of houses). Numbers represent totals pooled over study villages and the collection period (October 2016 to March 2017). [file 12936_2019_3030_MOESM6_ESM.docx]

**Additional Table S3:** Number of An. gambiae complex females that tested positive for the presence of P. falciparum sporozoites during dry (November 2016 - March 2017 and November - December 2017) and wet season (October 2016) with different trapping methods (HLC, MET) and at different trapping locations (indoors versus outside of houses). Numbers represent totals pooled over study villages and the collection period (October 2016 to March 2017).

|  |  | HLC | | MET | |  |  |
| --- | --- | --- | --- | --- | --- | --- | --- |
|  |  | Indoor | Outdoor | Indoor | Outdoor | Total | Total tested |
| Dry | *An. coluzzii* | 15 | 10 | 6 | 4 | 35 | 1089 |
|  | *An. gambiae* | 7 | 9 | 1 | 6 | 23 | 357 |
| Wet | *An. coluzzii* | 10 | 7 | 7 | 0 | 24 | 713 |
|  | *An. gambiae* | 23 | 30 | 11 | 11 | 75 | 875 |
|  | Total | 55 | 56 | 25 | 21 | 157 | 3191 |
