# Supplementary material for: Evaluation of mosquito electrocuting traps as a safe alternative to the human landing catch for measuring human exposure to malaria vectors in Burkina Faso
Source: Malar J. 2019 Dec 2;18:386. doi: 10.1186/s12936-019-3030-5 (PMC6889701; doi:10.1186/s12936-019-3030-5)
Supplement: Supplementary file 7 — Additional file 7. Number (raw data) of An. gambiae s.l. collected per month from (October 2016 to December 2017 by trapping methods a indoor and b outdoor using mosquito electrocuting trap (MET) and human landing catch (HLC). [file 12936_2019_3030_MOESM7_ESM.pptx]

## Slide 1
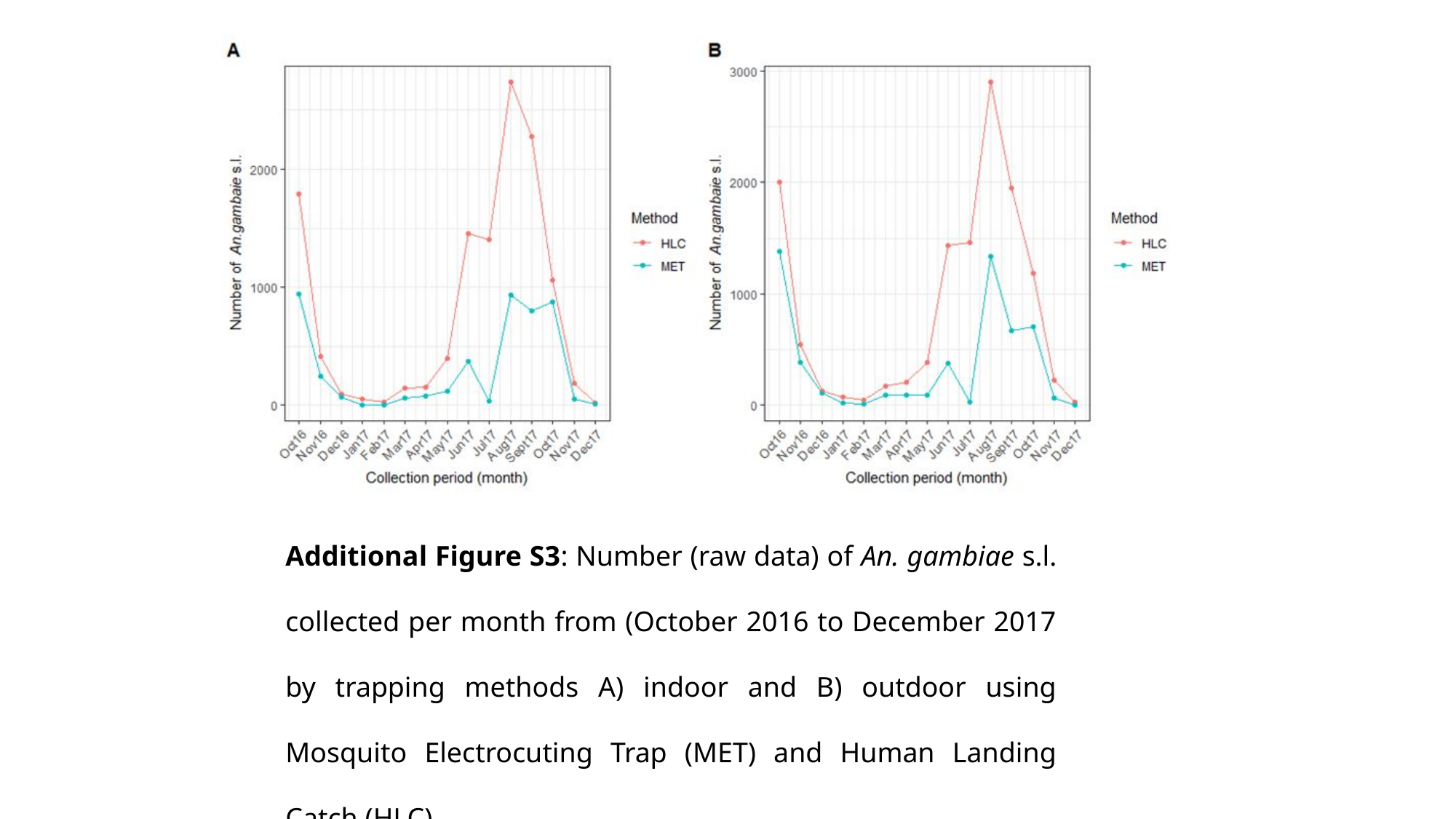

Additional Figure S3: Number (raw data) of An. gambiae s.l. collected per month from (October 2016 to December 2017 by trapping methods A) indoor and B) outdoor using Mosquito Electrocuting Trap (MET) and Human Landing Catch (HLC).
