# Supplementary material for: Evaluation of mosquito electrocuting traps as a safe alternative to the human landing catch for measuring human exposure to malaria vectors in Burkina Faso
Source: Malar J. 2019 Dec 2;18:386. doi: 10.1186/s12936-019-3030-5 (PMC6889701; doi:10.1186/s12936-019-3030-5)
Supplement: Supplementary file 8 — Additional file 8. Range of average temperature (°C) and relative humidity (%) recorded at the mosquito collection point using data logger. [file 12936_2019_3030_MOESM8_ESM.docx]

**Additional Table S4**: Range of average temperature (°C) and relative humidity (%) recorded at the mosquito collection point using data logger.

|  | Temperature (C) | Relative humidity (%) |
| --- | --- | --- |
| Indoor | 27.6 (17 - 37.24) | 58.62 (15.11 - 99.9) |
| Outdoor | 25.16 (15.7 - 3884) | 64.06 (11.73 - 99.95) |
