# Supplementary material for: Evaluation of mosquito electrocuting traps as a safe alternative to the human landing catch for measuring human exposure to malaria vectors in Burkina Faso
Source: Malar J. 2019 Dec 2;18:386. doi: 10.1186/s12936-019-3030-5 (PMC6889701; doi:10.1186/s12936-019-3030-5)
Supplement: Supplementary file 9 — Additional file 9. Effect of the humidity on the mean predicted number of An. gambiae s.l. collected per night according over the trapping methods, location and village. The solid black line indicates the regression line based on the model and grey-shaded area indicates the 95% CIs. Humidity data were only available for part of the sampling period (e.g mostly during the dry season months [Nov 2016 to April 2017, and Nov to Dec 2017], and a few months in wet season [October 2016 and May to October 2017]. The predicted relationship between relative humidity and vector abundance is thus based on months in which matched data were available. [file 12936_2019_3030_MOESM9_ESM.pptx]

## Slide 1
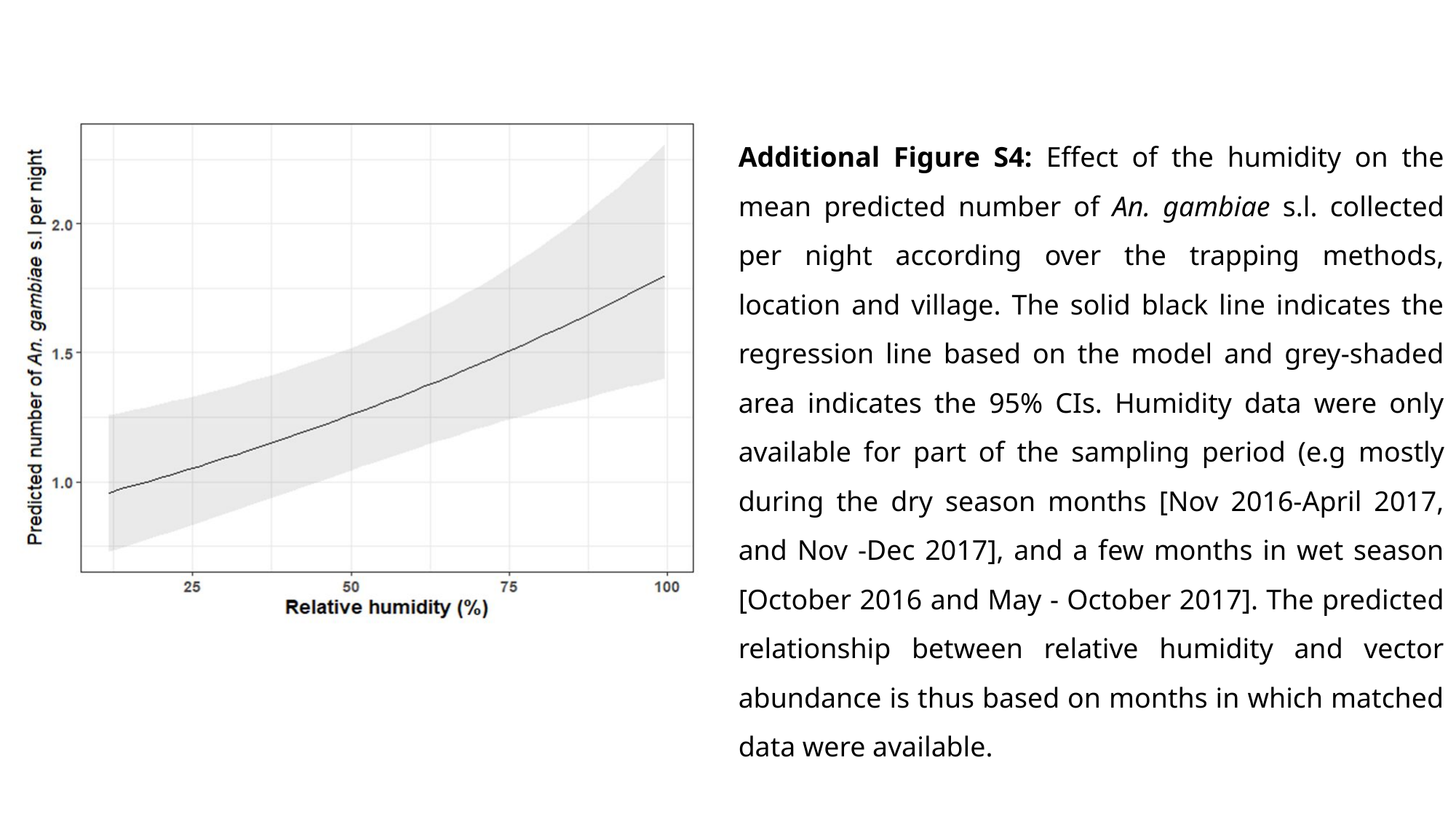

Additional Figure S4: Effect of the humidity on the mean predicted number of An. gambiae s.l. collected per night according over the trapping methods, location and village. The solid black line indicates the regression line based on the model and grey-shaded area indicates the 95% CIs. Humidity data were only available for part of the sampling period (e.g mostly during the dry season months [Nov 2016-April 2017, and Nov -Dec 2017], and a few months in wet season [October 2016 and May - October 2017]. The predicted relationship between relative humidity and vector abundance is thus based on months in which matched data were available.
