# Supplementary material for: Evaluation of mosquito electrocuting traps as a safe alternative to the human landing catch for measuring human exposure to malaria vectors in Burkina Faso
Source: Malar J. 2019 Dec 2;18:386. doi: 10.1186/s12936-019-3030-5 (PMC6889701; doi:10.1186/s12936-019-3030-5)
Supplement: Supplementary file 10 — Additional file 10. Mean predicted number of An. gambiae s.l. collected per night and season over the trapping methods, location and village with 95% CIs. Dry season indicates An. gambiae s.l. collected from November to April whilst wet season corresponds to period between May and October. [file 12936_2019_3030_MOESM10_ESM.pptx]

## Slide 1
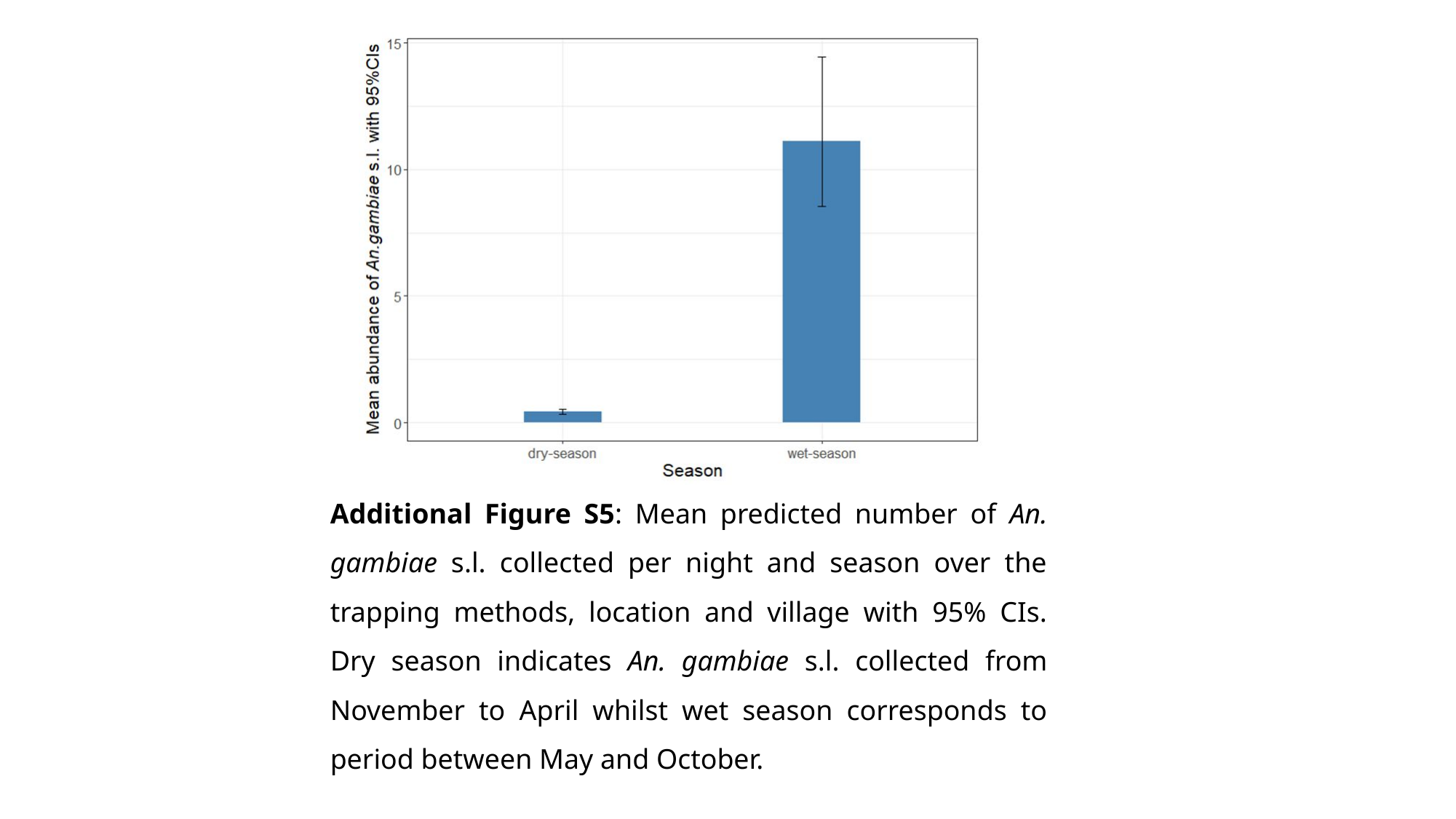

Additional Figure S5: Mean predicted number of An. gambiae s.l. collected per night and season over the trapping methods, location and village with 95% CIs. Dry season indicates An. gambiae s.l. collected from November to April whilst wet season corresponds to period between May and October.
