# Supplementary material for: Evaluation of mosquito electrocuting traps as a safe alternative to the human landing catch for measuring human exposure to malaria vectors in Burkina Faso
Source: Malar J. 2019 Dec 2;18:386. doi: 10.1186/s12936-019-3030-5 (PMC6889701; doi:10.1186/s12936-019-3030-5)
Supplement: Supplementary file 11 — Additional file 11. Mean predicted proportion of An. coluzzii relative to An. gambiae collected per village from October 2016 to December 2017, pooled over the trapping location and methods, with 95% CIs. [file 12936_2019_3030_MOESM11_ESM.pptx]

## Slide 1
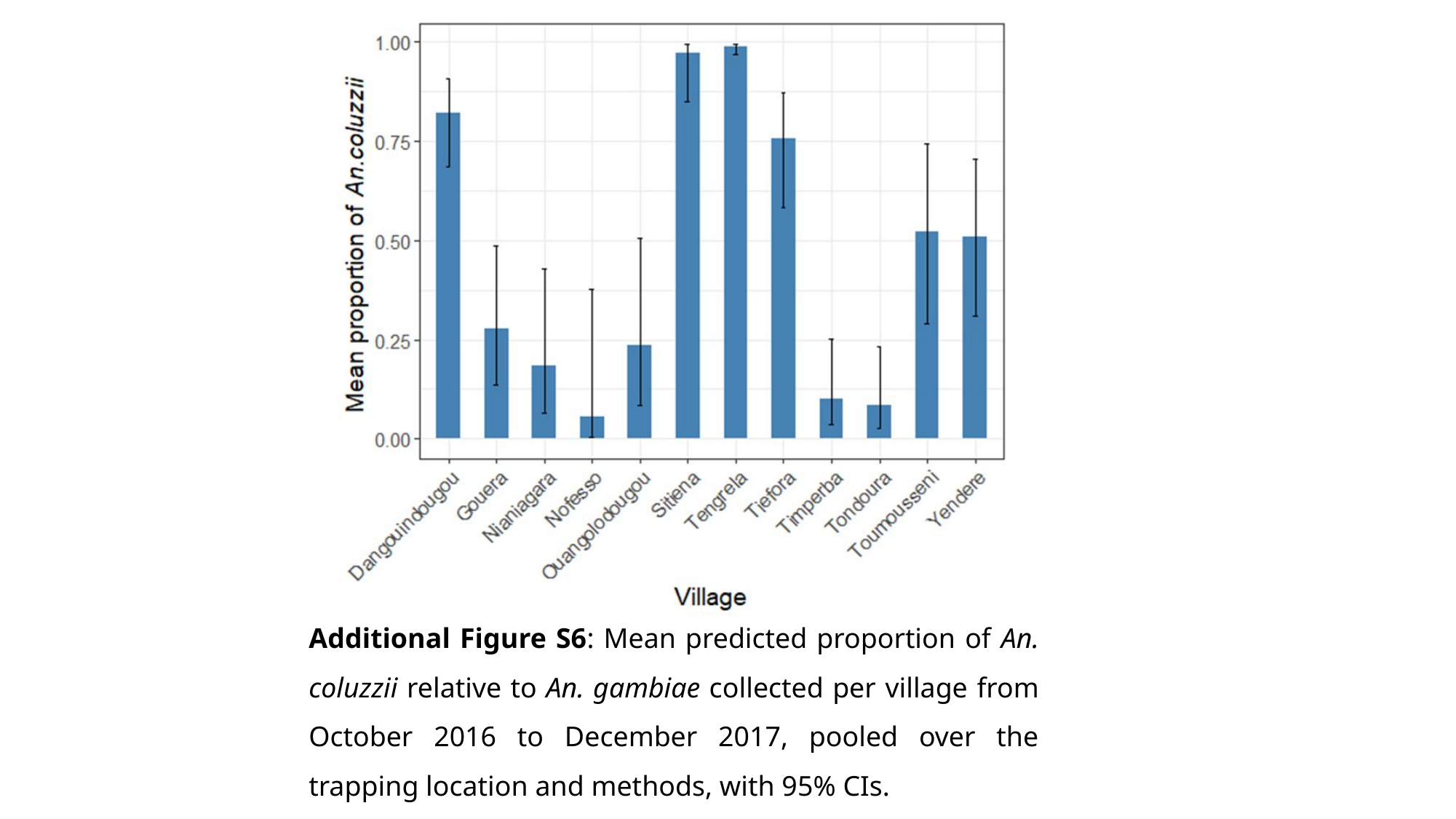

Additional Figure S6: Mean predicted proportion of An. coluzzii relative to An. gambiae collected per village from October 2016 to December 2017, pooled over the trapping location and methods, with 95% CIs.
