# Supplementary material for: Evaluation of mosquito electrocuting traps as a safe alternative to the human landing catch for measuring human exposure to malaria vectors in Burkina Faso
Source: Malar J. 2019 Dec 2;18:386. doi: 10.1186/s12936-019-3030-5 (PMC6889701; doi:10.1186/s12936-019-3030-5)
Supplement: Supplementary file 12 — Additional file 12. Effect of the mean relative humidity on the estimation of the proportion of An. coluzzii. The solid black line is the regression line of the predicted proportions and the grey-shaded area indicate the 95% CIs. [file 12936_2019_3030_MOESM12_ESM.pptx]

## Slide 1
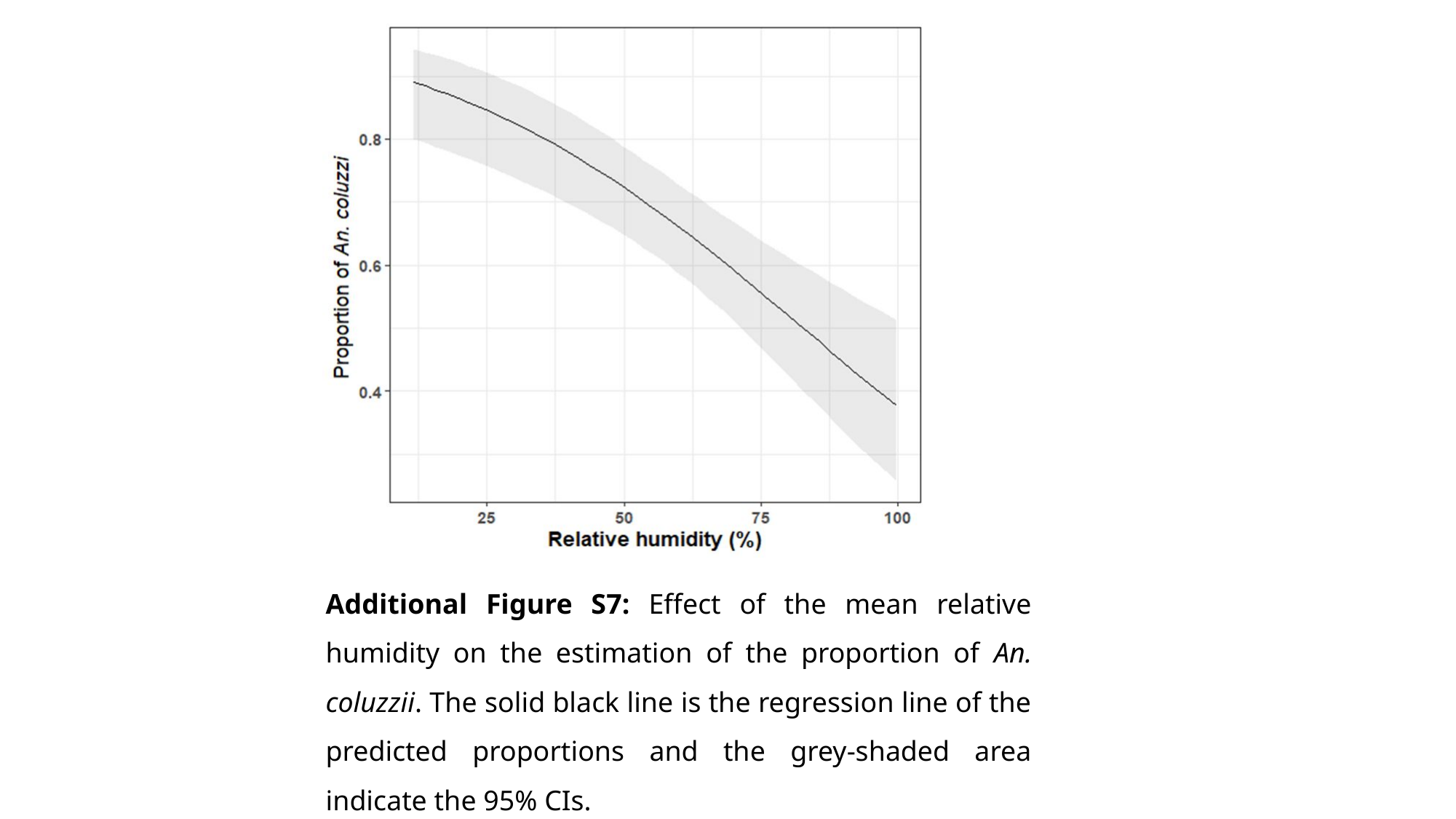

Additional Figure S7: Effect of the mean relative humidity on the estimation of the proportion of An. coluzzii. The solid black line is the regression line of the predicted proportions and the grey-shaded area indicate the 95% CIs.
