# Supplementary material for: Evaluation of mosquito electrocuting traps as a safe alternative to the human landing catch for measuring human exposure to malaria vectors in Burkina Faso
Source: Malar J. 2019 Dec 2;18:386. doi: 10.1186/s12936-019-3030-5 (PMC6889701; doi:10.1186/s12936-019-3030-5)
Supplement: Supplementary file 13 — Additional file 13. Mean predicted Plasmodium falciparum infection rate in An. gambiae s.l. collected per village from October 2016 to December 2017, pooled over the trapping location and methods, with 95% CIs. [file 12936_2019_3030_MOESM13_ESM.pptx]

## Slide 1
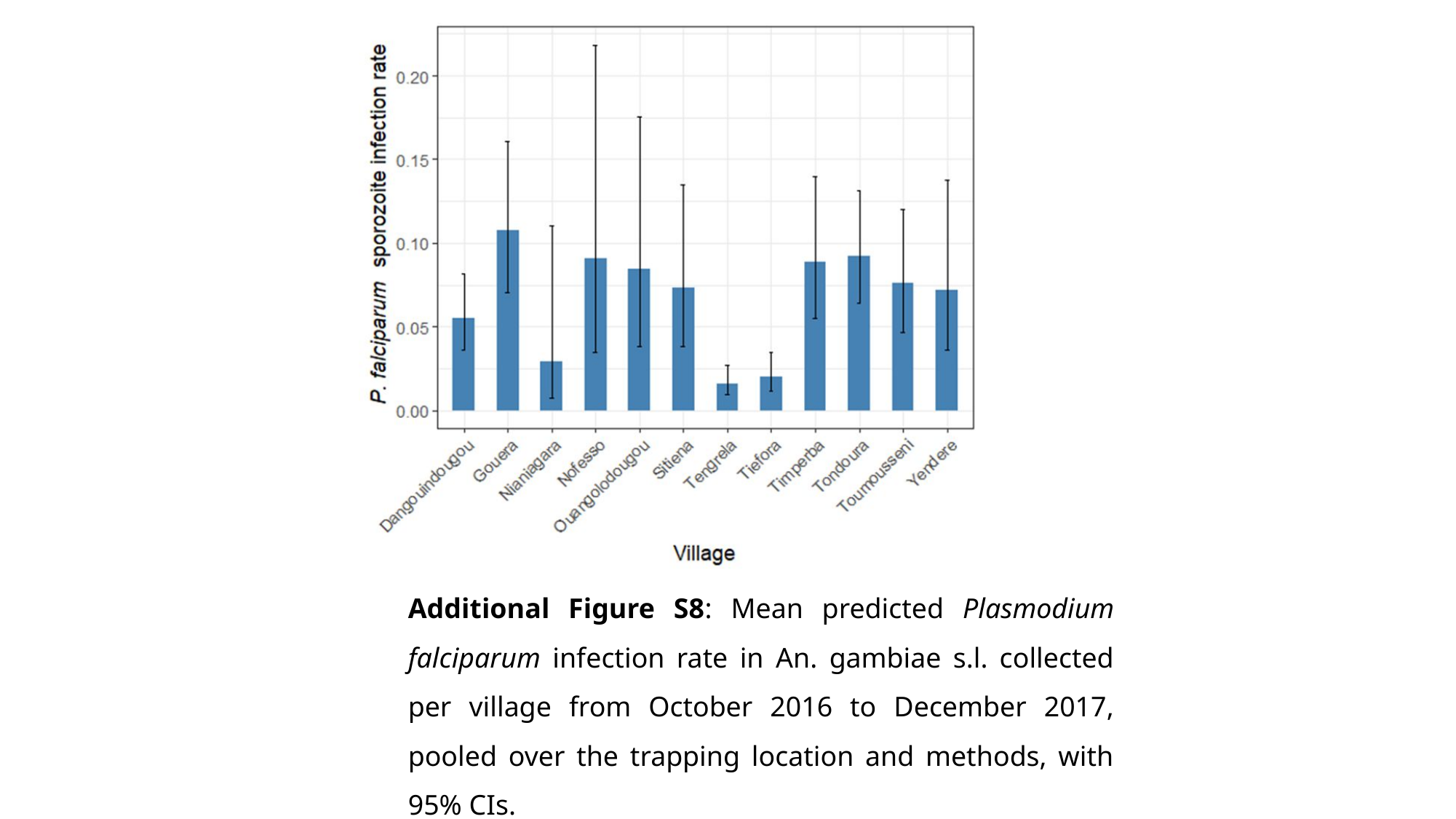

Additional Figure S8: Mean predicted Plasmodium falciparum infection rate in An. gambiae s.l. collected per village from October 2016 to December 2017, pooled over the trapping location and methods, with 95% CIs.
