# Supplementary material for: Evaluation of mosquito electrocuting traps as a safe alternative to the human landing catch for measuring human exposure to malaria vectors in Burkina Faso
Source: Malar J. 2019 Dec 2;18:386. doi: 10.1186/s12936-019-3030-5 (PMC6889701; doi:10.1186/s12936-019-3030-5)
Supplement: Supplementary file 14 — Additional file 14. Non-significant term excluded from the best models where LRT represents likelihood ratio test and degree of freedom of 1 for all the terms. [file 12936_2019_3030_MOESM14_ESM.docx]

**Additional Table S5:** Non-significant term excluded from the best models where LRT represents Likelihood Ratio Test and degree of freedom of 1 for all the terms.

|  | Season: Method | |  | Scale (Humidity) | |  | Scale (temperature) | |
| --- | --- | --- | --- | --- | --- | --- | --- | --- |
|  | LRT | p-values |  | LRT | p-values |  | LRT | p-values |
| Proportion of indoor biting (P_i_) | 0.31 | 0.57 |  | 0.21 | 0.64 |  | 0.02 | 0.87 |
| Proportion caught when most people are indoors (P_fl_) | 0.28 | 0.60 |  | 0.33 | 0.56 |  | 0.75 | 0.38 |
| Proportion of human exposure occurring indoors (π_i_) | 0.02 | 0.30 |  | 0.20 | 0.65 |  | 0.03 | 0.84 |
